# Self-regulation of functional pathways by motifs inside the disordered tails of beta-catenin

**DOI:** 10.1186/s12864-016-2825-9

**Published:** 2016-08-31

**Authors:** Bi Zhao, Bin Xue

**Affiliations:** Department of Cell Biology, Microbiology and Molecular Biology, School of Natural Sciences and Mathematics, College of Arts and Sciences, University of South Florida, 4202 E. Fowler Ave, ISA 2015, Tampa, 33620 FL USA

**Keywords:** Beta-Catenin, Wnt signaling pathway, Cadherin junction formation, Intrinsic disorder, Auto-regulation

## Abstract

**Background:**

Beta-catenin has two major functions: coordinating cell-cell adhesion by interacting with cadherin in cadherin junction formation pathway; and regulating gene expression through Wnt signaling pathway. Accomplishing these two functions requires synergistic action of various sequential regions of the same beta-Catenin molecule, including the N-terminal tail, the middle armadillo domain, and the C-terminal tail. Although the middle armadillo domain is the major functional unit of beta-Catenin, the involvement of tails in the regulation of interaction between beta-Catenin and its partners has been well observed. Nonetheless, the regulatory processes of both tails are still elusive. In addition, it is interesting to note that the three sequential regions have different structural features: The middle armadillo domain is structured, but both N- and C-terminal tails are disordered. This observation leads to another important question on the functions and mechanisms of disordered tails, which is also largely unknown.

**Results:**

In this study, we focused on the characterization of sequential, structural, and functional features of the disordered tails of beta-Catenin. We identified multiple functional motifs and conserved sequence motifs in the disordered tails, discovered the correlation between cancer-associated mutations and functional motifs, explored the abundance of protein intrinsic disorder in the interactomes of beta-Catenin, and elaborated a working model on the regulatory roles of disordered tails in the functional pathways of beta-Catenin.

**Conclusion:**

Disordered tails of beta-Catenin contain multiple functional motifs. These motifs interact with each other and the armadillo domain of beta-catenin to regulate the function of beta-Catenin in both cadherin junction formation pathway and Wnt signaling pathway.

**Electronic supplementary material:**

The online version of this article (doi:10.1186/s12864-016-2825-9) contains supplementary material, which is available to authorized users.

## Background

Beta-Catenin is a 92-kDa protein that is composed of two flexible tails at each of the N- and C-termini, and an intermediate structured armadillo domain (ARM) containing 12 repeats of helical segments [1]. The function of beta-Catenin is to regulate cadherin junction formation [2, 3] and to regulate Wnt signaling [4, 5]. Therefore, beta-Catenin plays critical roles in many biological processes, such as embryonic development [6, 7], cell division [8], and maintenance of pluripotency [9]. Disorganized expression of beta-Catenin is associated with many diseases, including cancer [10–12] and cardiovascular diseases [13, 14]. The ARM domain is the major determinant for the function of beta-Catenin. It binds to the cytoplasmatic tail of cadherin at the stage of junction formation. ARM domain also interacts with short motifs on both Axis Inhibition Protein (Axin) and Adenomatous Polyposis Coli tumor repressor protein (APC) in Wnt signaling pathway [15]. The interaction raises the local concentration of beta-Catenin and leads to the phosphorylation, ubiquitination, and degradation of beta-Catenin [16]. Upon the activation of Wnt signaling molecules, beta-Catenin molecules escape from the degradation pathway, accumulate in cytoplasma, and translocate into nucleus. One possible translocation mechanism involves the interaction between the ARM domain and the phenylalanine-glycine(FG)-repeat containing proteins in the Nuclear Pore Complex (NPC) [17, 18]. Once accessing the nucleus, beta-Catenin uses its ARM domain to interact with T Cell Factor/Lymphoid Enhancer Factor (TCF/LEF) family transcription factors to activate downstream gene expression [19–21].

The disordered tails of beta-Catenin were also found to synergistically regulate the function of beta-Catenin in recent studies. The tails regulate the binding between beta-Catenin and cadherin [22]. The N-terminal tail of beta-Catenin is also phosphorylated by the destruction complex, which is formed by Axin, APC, Casein Kinase I alpha (CK1-alpha), Glycogen Synthase Kinase 3β (GSK-3beta), and Protein Phosphatase 2A (PP2A) [23–25]. Phosphorylated beta-Catenin is ubiquitinated and then degraded [26–28]. Wnt signaling molecules outside of the cell are able to prevent the formation of destruction complex and therefore increase the cytoplasmic level of beta-Catenin. In this way, the cytoplasmic level of beta-Catenin is tightly regulated. The flexible tail of beta-Catenin also facilitates its nuclear translocation [29, 30], and recruits factors involved in transcription activation [31].

These discoveries have extended our comprehension on the interaction patterns between beta-Catenin and its partners, and opened a new field on the functional roles of flexible tails. However, the detailed mechanisms associated with these new discoveries are still largely unknown. In this study, we applied systematic bioinformatics analyses on both N- and C-terminal tails of beta-Catenin, identified multiple functional motifs, characterized the conserved sequential segments, discovered the pattern of interactions between beta-Catenin and its partners, revealed the correlation between cancer-associated mutations and functional motifs, and proposed possible regulatory mechanisms of flexible tails on the function of beta-Catenin. This study increases our knowledgebase on the sequential, structural, and functional features of beta-Catenin tails, facilitates our understanding on the mechanisms and regulatory roles of the terminal tails on the functions of beta-Catenin.

## Method

### Sequence analysis

**(1) Sequence conservation**: The FASTA sequences of beta-Catenin from different species were extracted from UniprotKB [32]. The sequences were used as input of different predictors. The sequences were also aligned and uploaded to WebLoGo [33] to generate sequence logo plots to demonstrate the conserved patterns of amino acids. **(2) Mutation analysis:** Amino acid substitutions in human beta-Catenin were retrieved from UniprotKB, COSMIC (Catalogue of Somatic Mutation in Cancer) [34], DMDM (Domain Mapping of Disease Mutations) [35], and BioMuta [36] databases. These four databases have 20, 278, 20, and 138 mutations, respectively. Both of the UniProtKB and DMDM databases contain one unique mutation that is not found in other databases. COSMIC and BioMuta have 128 overlapped mutations. The total number of unique mutations is 281. These mutations were then analyzed in parallel with functional sites/motifs obtained from motif analysis.

### Structural analysis

**(1) Secondary structure prediction:** NetSurfP [37] was used to predict the secondary structure of beta-Catenin. The NetSurfP predictor applied a two-step strategy, of which the first step was used to predict the secondary structure of amino acids in three states (helix, strand, and coil) by using protein sequence as input, and the second step was a filter by taking the predicted secondary structures of the first step over a sliding window as input. In both steps, artificial neural networks were applied to make predictive output based on the input data. **(2) Disorder prediction:** Both PONDR-FIT [38] and PONDR©VLXT [39] were applied to predict per-residue disorder score of beta-catenin. PONDR-FIT is an artificial neural network based meta-predictor and is composed of six individual predictors: PONDR©VLXT, PONDR-VSL2 [40], PONDR-VL3 [41], FoldIndex [42], IUPred [43], and TopIDP [44]. Since the integration of individual predictors in the meta-strategy combines all the possible correct predictions, the accuracy of meta-predictor is normally improved significantly. In fact, PONDR-FIT is one of the most accurate predictors for protein intrinsic disorder [45]. PONDR©VLXT is highly efficient in identifying hydrophobic motifs within long disordered regions. The input features of PONDR©VLXT include various combinations of amino acids, mainly hydrophobic residues. For this reason, PONDR©VLXT is very sensitive to the local changes of amino acids and can be used to easily detect local hydrophobic clusters. In the outputs of both predictors, residues with score higher than 0.5 were considered to be disordered, residues with score lower than 0.5 were treated as structured. PONDR-FIT was also used to analyze proteins in the primary and the secondary interactomes of beta-Catenin, as well as proteins in the related signaling pathways.

### Functional analysis

**(1) Binding Motifs:** ANCHOR [46], MoRF [47], and MoRFpred [48] were used to predict potential binding motifs inside disordered regions. ANCHOR identifies binding motifs that are dependent on hydrophobic clusters, MoRF is able to detect binding motifs that undergo structural transition from coil to helix, and MoRFpred finds binding motifs that change conformations from coil to helix, strand, and other types of coils. **(2) Linear Motifs:** ELM [49] online server was applied to predict possible functional linear motifs. ELM contains short linear motifs that normally stretch over three to ten residues. The ELM motifs have broad functions, including cleavage, binding, modification, etc. **(3) Protein Interaction Networks and Pathways:** The protein-protein interaction networks were collected from the STRING database [50] by using confidence score 0.85. STRING is one of the most comprehensive databases for protein-protein interactions. Proteins signaling pathways were extracted from KEGG [51], which is a prevailing tool for the scientific community. The gene names/protein IDs from STIRNG and KEGG were also used to extract corresponding protein sequences from UniProtKB. **(4) FATHMM** (Functional Analysis Through Hidden Markov Models) [52]: FATHMM is an advanced high-throughput predictor trained by using hidden Markov model on multiple mutation databases for analyzing the functional impacts of mutations. FATHMM outperformed many other predictors on multiple validation datasets. All the mutations were then classified by FATHMM using two independent variables (criteria): Damaging (or Tolerant) and Cancer-promoting (or non-Cancer-promoting), into four groups: Damaging and Cancer-promoting, Damaging and non-Cancer-promoting, Tolerant and Cancer-promoting, and Tolerant and non-Cancer-promoting.

## Results

### Disordered tails at both N- and C-termini accommodate predicted binding motifs

**Fig. 1:**
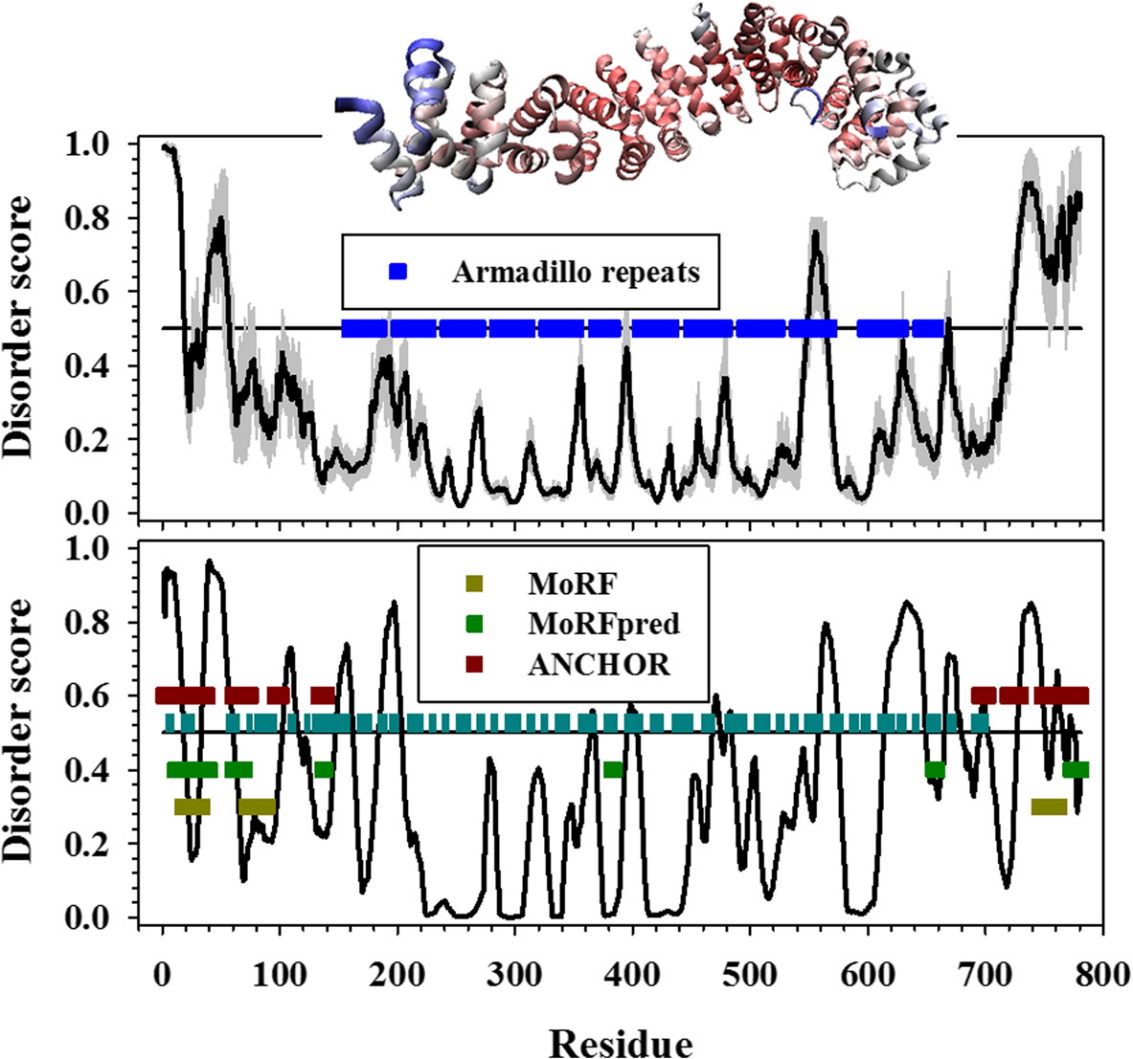
Predicted intrinsic disorder of and predicted binding motifs in human beta-Catenin (UniProtKB Entry: P35222). X-axis shows sequential indexes of amino acids. Y-axis displays disorder scores predicted by PONDR-FIT (upper panel) and PONDR©VLXT (lower panel). Residues with score higher than 0.5 are disordered, otherwise structured. In addition, residues with score between ~0.3 and 0.5 are assumed to be flexible, and residues with score close to zero are extremely rigid. The gray shadow behind PONDR-FIT prediction is the estimated prediction error from PONDR-FIT predictor. Horizontal blue bars in the upper panel represent the domain structure of twelve armadillo repeats. Dark cyan bars in the middle of lower panel are helices predicted by NetSurP. Binding motifs were predicted by MoRF (dark yellow), ANCHOR (brown), and MoRFpred (dark green) predictors, respectively. The 3D structure for the armadillo domain of human beta-Catenin (PDB id: 1JDH) was placed on the top of the figure

PONDR-FIT [38], which is one of the most accurate predictors of protein intrinsic disorder [45], was applied to identify disordered regions of beta-Catenin. The prediction of PONDR-FIT as shown in the upper panel of Fig. 1 demonstrates that: (1) the middle region of human beta-Catenin from ~ AA130 to ~ AA710 is structured; and (2) the N- and C-terminal tails flanking the middle region are disordered. These conclusions are supported by the prediction of PONDR©VLXT [39] predictor as shown in the lower panel of the same figure. The disorder curve of PONDR©VLXT prediction is more fluctuating due to the fact that this predictor is very sensitive to local composition of amino acids. Even though, the disorder scores of the middle region from ~ AA220 to ~ AA600 are much lower than that of the flanking tails, indicating the conformation of the middle region is much more rigid than the flanking tails. In addition to the consistency between different computational predictors, the conclusions on structural flexibility of beta-Catenin are also supported by experimental results. In a recent x-ray crystallography study, the structure of beta-Catenin was resolved for a region spanning from AA138 to AA691 [1], which is similar to the above-mentioned structured region identified by both PONDR-FIT and PONDR©VLXT. This region contains an armadillo domain of which the structure is determined to be twelve repeats of helical segments as shown on the top of Fig. 1. Regions other than this armadillo domain failed to be crystallized, indicating the presence of considerable structural flexibility in these regions. These results come to a consistent conclusion that the armadillo domain has inflexible 3D structure, while both tails are disordered. For the simplicity of discussion in this study, N-terminal and C-terminal tails of human beta-Catenin were defined as AA1-AA150 and AA667-AA781, respectively.

The lower panel of Fig. 1 shows: (1) the disorder prediction of PONDR©VLXT predictor, (2) the secondary structure prediction from NetSurfP [53], which is one of the most accurate and popular predictors of protein secondary structure, and (3) binding motifs identified by Molecular Recognition Feature (MoRF) [47], MoRFpred [48], and ANCHOR [46], respectively. MoRF predicts binding motifs that change their conformations from coils to helices upon binding, MoRFpred identifies binding motifs that transform from coil into one of the three basic secondary structures (helix, strand, and coil), and ANCHOR recognizes interaction motifs dominated by hydrophobic residues. The disorder curves of PONDR©VLXT prediction at both tails display multiple “dip” regions, which indicate hydrophobic clusters of amino acids inside disordered regions. The segments corresponding to these dips often function as binding motifs. Secondary structure prediction demonstrates that the central ARM domain is composed of many helical segments, the N-terminal tail has multiple scattered helical segments, but the C-terminal tail has only a couple of helical segments in the first ~40 residues and is depleted of helices in the rest ~80 residues. The predicted binding motifs were shown as short bars in the lower panel of Fig. 1. At the N-terminal end (AA1 ~ AA150) of the disorder curve, all three dips (at ~ AA25, ~AA80, and ~ AA130, respectively) are overlapped with binding motifs predicted by at least two predictors. At the C-terminal end (over ~ AA660), the segment corresponding to a composite dip at ~ AA760 was predicted to be a binding motif by three predictors. Another two segments next to the dip at ~ AA720 were predicted to be binding motifs by ANCHOR. The forth segment overlapped with the dip at ~ AA660 was predicted as binding motif by MoRFpred. All the predicted binding motifs, except the last two (~AA720 and ~ AA760) that locate at the very end of C-terminal tail, are also predicted to be helices.

### Disordered tails are enriched of linear motifs

**Fig. 2:**
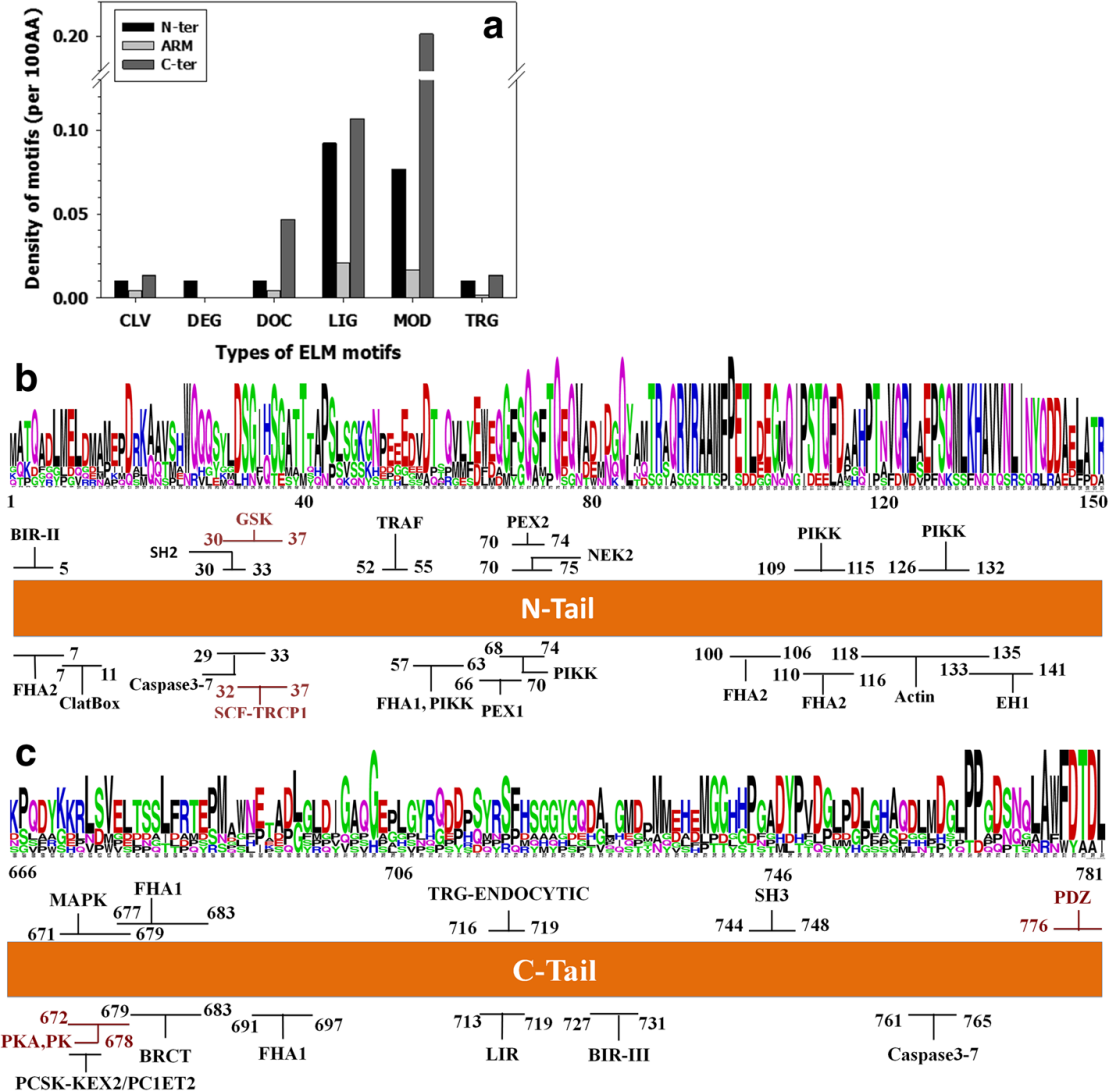
ELM motifs in beta-Catenin. **a** Distribution of six types of ELM motifs in the N-terminal tail, the ARM domain, and the C-terminal tail. The six types of ELM motifs are: CLV–cleavage site, DEG–degragon, DOC–modifying enzyme docking, LIG–ligand binding, MOD–post-translational modification site, and TRG–subcellular targeting. The density of each type of ELM motif in each region of beta-Catenin was calculated by firstly normalizing the number of a specific type of motifs in that region over the total number of ELM motifs in beta-Catenin, and then scaling the result over the length of that region based on every 100 residues. **b** and **c** demonstrate the locations and conservation of ELM motifs in the N- and C-terminal tails, respectively. Motifs in red are experimentally validated. In both figures, “redundant” motifs that belong to the same functional family or overlap with each other on their amino acid sequences have been filtered out to keep the clarity and simplicity of the figures. The weblogo plots were built using beta-Catenin sequences from eight species (Human, Mouse, Bovine, Dog, Turtle, Frog, Jelly fish, and Urchin, see Additional file 2: Table S2)

Eukaryotic Linear Motifs (ELMs) are short sequence motifs of normally several amino acids, but perform multiple types of functions [49]. By searching ELM database [49], 130 ELMs were identified in beta-Catenin. In which, 73 ELMs locate in the tail regions (Additional file 1: Table S1). Figure 2a shows the density of each type of ELMs in the middle domain and the tail regions of beta-Catenin. It is clear that the densities of different types of functional motifs in both tails are much higher than that in the ARM domain. Both tails are extremely enriched of MOD and LIG motifs. In addition, while only the N-terminal tail has DEG motifs, the C-terminal tail has many more MOD and DOC motifs. To demonstrate the sequential distribution of motifs, the locations of a non-redundant set of ELM motifs in the tail regions were labeled for both N-terminal and C-terminal tails in Fig. 2b and c. At the N-terminal tail, ELM motifs cluster at three locations: ~AA20, ~AA70, and ~ AA120. In the C-terminal tail, the motifs congregate at ~ AA675, ~AA720, and ~ AA760. Clearly, these locations are essentially the same as the dips and binding motifs predicted in Fig. 1.

Among these predicted motifs, six have been experimentally validated: The MOD_GSK3_1 motif (AA30-37) is phosphorylated by GSK3-beta [54–56]; The ubiquitin ligase binding motif DEG_SCF_TRCP1_1 (AA32-37) recognizes F box in SCF (beta-TrCP1) for subsequent ubiquitination [57]; DOC_WW_Pin1_4 (AA243-248) on beta-Catenin interacts with prolyl somerase Pin1 [53]; MOD_PKA_1 motifs (AA549-555 and AA672-678) are phosphorylated by PKA kinase [58]; and the PDZ-binding motif LIG_PDZ_Class_1 (AA776-781) forms complex with TIP-1 [59].

Weblogo analyses in Fig. 2b and c present multiple sequential patterns and sequence motifs that are conserved cross eight species (Additional file 2: Table S2). Most notably, AA90 ~ AA130 at N-terminal tail and AA774 ~ 781 at the very end of C-terminal tail accommodate highly conserved motifs. Other regions may not contain highly conserved consecutive amino acids sequences, but do have highly conserved patterns of amino acids. To name a few, both AA670 ~ AA680 and AA730 ~ AA740 have conserved hydrophobic patterns. Although being conserved, many of these segments are still lack of functional annotations, such as AA90 ~ AA100, AA140 ~ AA150, AA730 ~ AA740, and AA750 ~ AA760.

### N-terminal tail is associated with the majority of sequential variances

**Fig. 3:**
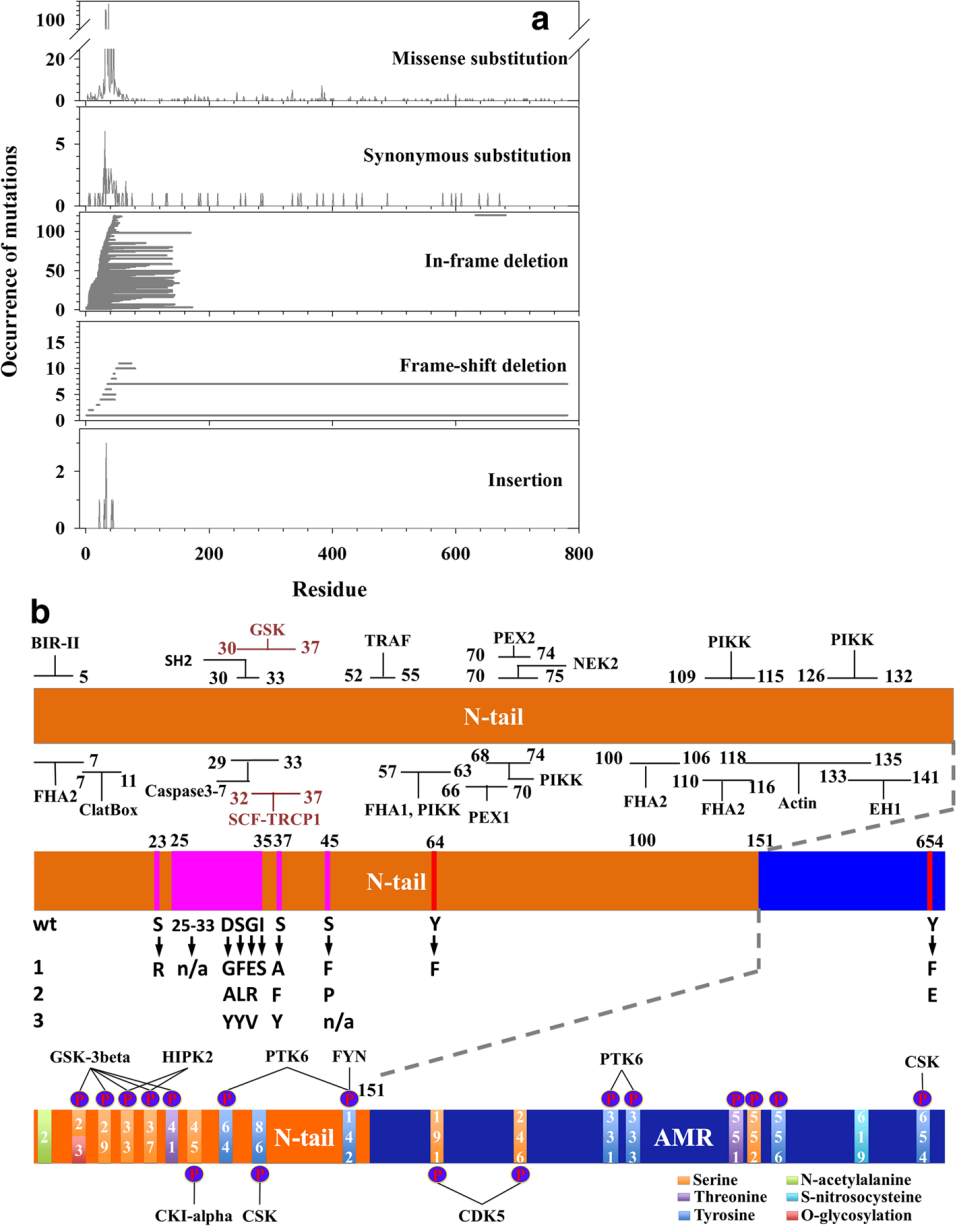
Sequence variances in disordered tails. Information on the mutation and modification of beta-Catenin was extracted from UniProtKB, COSMIC, BioMute, and DMBM. **a** Occurrences of five different types of mutations. The types of mutations from top to bottom are: (1) missense mutation, (2) synonymous mutation, (3) in-frame deletion, (4) frameshift deletion, and (5) insertion. X-axis shows the amino acid sequence. Y-axis is the occurrences of mutations. The plots in (1), (2), and (5) show the number of point mutations at each site. The lines in (3) and (4) represent deleted segments. **b** Mutations and post-translational modifications in the N-terminal tail of beta-Catenin. The upper inset shows a non-redundant set of functional motifs at the N-terminal tail of beta-Catenin. The middle inset demonstrates mutations from UniProtKB for the N-terminal tail and Tyr-654 near the C-terminal tail. Red bars stand for natural variants and pink bars for mutagenesis sites. The character label “wt” and the numbers at the left-hand side indicate wild type (wt) and mutations (1, 2, and 3). Lower inset shows observed post-translational modifications in both N- and C-terminal tails

Figure 3a shows the sequential distribution of mutations of beta-Catenin collected from four databases, including UniProtKB [32], COSMIC [34], DMDM [35], and BioMuta [36]. Clearly, almost all the deletions and insertions are only observed at the first half of the N-terminal tail (AA1 ~ AA75). The majority of synonymous and missense mutations are also in the first half of the N-terminal tail, although a small portion do spread along the entire sequence. More specifically, over 82 % of the mutations occur in the region from AA1 to AA75, and over 71 % of the mutations present in a smaller segment from AA25 to AA75.

Figure 3b presents the mutations and modifications on both N- and C-terminal tails of beta-Catenin extracted from UniProtKB, together with the functional motifs predicted from ELM. The UniProtKB information on mutation and modification is of high confidence due to the fact that the information is either experimentally validated or observed on their homologous sequences. By comparing the locations of these mutations and modifications, it is clear that most of them are in the region from AA25 ~ AA50. The involved amino acid residues include Ser-23, 33, 37, 45, Trp-25, Asp-32, Gly-34, Ile-35, and deletion of N’-WQQQSYLDS-C’ (AA25-33). These mutations overlap with one CLV, two DEG, one DOC, eight LIG, 12 MOD, and two TRG ELMs (Additional file 1: Table S1). In terms of functional influences, Ser-45, Ser-37, and Ser-33 at the N-terminal tail are three critical residues of which the phosphorylation and subsequent ubiquitination lead to the degradation of beta-Catenin [23, 26–28]. Near the C-terminal tail, several mutations are observed on Tyr-654, which is also a critical residue that uses phosphorylation to regulate the interaction between beta-Catenin and cadherin [22, 60].

**Fig. 4:**
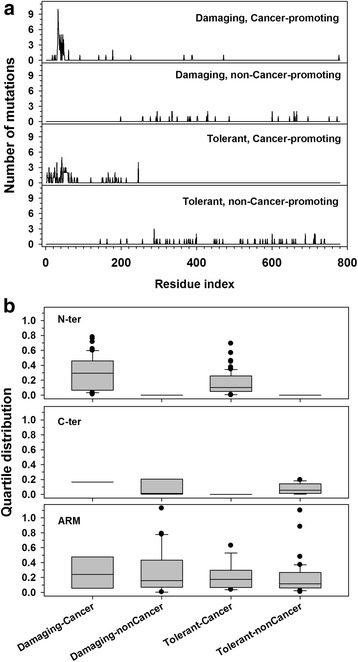
Correlation between functional variations and point mutations at different regions. By using FATHMM, all the mutations were assigned into one of the four groups based on two independent criteria: Damaging (or Tolerant) and Cancer-promoting (or non-Cancer-promoting). **a** Distribution of point mutations along the amino acid sequence of beta-Catenin in four groups (from top to bottom): Damaging and Cancer-promoting; Damaging and non-Cancer-promoting; Tolerant and Cancer-promoting; and Tolerant and non-Cancer-promoting. Y-axis shows the frequency of mutations at each sequential site. **b** Quartile distribution plot on the variation of structural flexibility caused by point mutation in afore-mentioned four groups of mutations (x-axis) for three different regions of beta-Catenin (top: the N-ter tail; middle: the C-ter tail; and bottom: the ARM domain). The structural flexibility was characterized using disorder score predicted by PONDR-FIT. The variation of structural flexibility at a specific site was calculated as the ratio of the change of disorder score at that site caused by the mutation to the disorder score at the same site of the wild-type sequence

FATHMM was used to evaluate the functional impact of point mutations as shown in Fig. 4. Figure 4a shows the sequential distribution of all the point mutations in four groups. Clearly, almost all the cancer-promoting mutations are located in the N-terminal tail of beta-Catenin, and non-cancer-promoting mutations are accommodated in either the ARM domain or the C-terminal tail. Although seeming to be similar, the distributions of mutations in the first group (damaging and cancer-promoting) and the third group (tolerant and cancer-promoting), as well as the distribution of mutations in the second group (damaging and non-cancer-promoting) and the fourth group (tolerant and non-cancer-promoting) are significantly different (P-values of F-test are 2.4e-11 and 1.2e-8, respectively). In addition, the frequencies of mutations at various sites in the N-terminal tail in the first group (damaging and cancer-promoting) are several times higher than the frequencies in other groups and regions. Figure 4b presents the change of structural flexibility caused by point mutation at three different regions in four different groups. Again, from the top and middle panels, it can be seen that the mutations at the N-terminal tail are cancer-promoting and are associated with large variations of structural flexibility. Meanwhile, the mutations at the C-terminal tail are non-cancer-promoting and are demonstrated by very limited variations of structural flexibility. In the bottom panel, it is clear that the damaging associated mutations may have large variations of structural flexibility, but the tolerant-associated mutations have relatively small changes of structural flexibility.

### Structural flexibility in the interaction partners of beta-catenin

Beta-Catenin interacts with its partners using its structured ARM domain and/or its flexible tails. Out of a total of 24 PDB structures for complexes formed between beta-Catenin and its partners, five are formed between the tails of beta-Catenin and the structured domains of other proteins, 18 complexes are formed between the structured ARM domain of beta-Catenin and the short segments of other proteins, the last one complex is formed between the ARM domain of beta-Catenin and a structured domain of another protein LRH-1 (Additional file 3: Table S3). In the first five complexes, disordered tails of beta-Catenin are involved. In the next 18 complexes, the short segments of the partners are either disordered or inside disordered regions. Therefore, 23 out of 24 interactions between beta-Catenin and its partners involve intrinsically disordered regions.

**Fig. 5:**
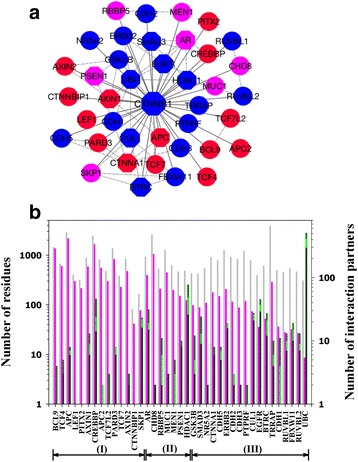
**a** Interaction partners of beta-Catenin (CTNNB1). The interaction partners were extracted from the STRING database using threshold value 0.85. The colors of the symbols indicate the fraction of disordered residues in that protein, categorized into three groups: blue (0–30 % of residues are intrinsically disorder), pink (30–50 %), and red (>50 %). The shapes of the symbols specify the number of interaction partners of that protein: Circle - less than five partners; Hexagon - more than five interaction partners. Lines between proteins imply that these two proteins interact with each other. **b** Abundance of intrinsic disorder in the secondary interactome of beta-Catenin. The secondary interactome refers to all the proteins that interact with one of the interaction partners of beta-Catenin. X-axis presents proteins in the first interactome shown in (A). All the proteins were ranked based on the fractions of disordered residues, with (I), (II), and (III) corresponding to the red, pink, and blue groups in (A), respectively. The first Y-axis on left-hand side shows the number of residues in each protein (gray bar) and the number of disordered residues in each protein (pink bar). The second Y-axis on the right-hand side uses stacked bars to show the distribution of intrinsic disorder in all the interaction partners of that protein indicated on X-axis. From bottom to top, the bars indicate number of proteins with 0–30 % of disordered residues (dark gray), number of proteins with 30–50 % of disordered residues (green), and number of proteins with more than 50 % of disordered residues (dark green). Both the first and the second Y-axes use base-10 log scale

To determine the abundance of protein intrinsic disorder in the protein interaction networks of beta-Catenin, we retrieved interaction partners of beta-Catenin from the STRING database [50] (Additional file 4: Table S4). STRING is a comprehensive database of protein-protein interactions with multiple choices of functional annotations and source of interactions. Figure 5a illustrates the distribution of protein intrinsic disorder in the interaction network of beta-Catenin. Out of 39 interaction partners of beta-Catenin, 14 proteins were predicted to be disordered, seven were predicted to carry large portion of disordered regions, and 18 were predicted to have small portion of disordered residues and therefore structured. Out of these 18 structured proteins, four cadherin-family members (CDH1, 2, 3, and 5) and a transcription factor LEF-1 use their flexible segments to bind the ARM domain of beta-Catenin (Additional file 3: Table S3). UBC, CUL1, FBXW11, and BTRC are proteins associated with ubiquitin ligase complex, which interacts with the N-terminal tail of beta-Catenin [28]. The other seven proteins are kinases, phosphatases, and deacetylase. These enzymes frequently target amino acids on the tails of beta-Catenin. In fact, as shown in Fig. 3, two groups of amino acids on or near the flexible tails of beta-Catenin undergo critical modifications: one group includes Ser-45, Thr-41, Ser-37, and Ser-33, which can be phosphorylated by CKI-alpha and GSK-3beta; the other group contains Tyr-654 that can be phosphorylated by CSK. The modifications on these two groups of amino acids regulate the function of beta-Catenin in the Wnt signaling pathway and the cadherin junction formation pathway, respectively [22, 23, 26–28, 60]. This observation agrees to the conclusions of our previous studies in which structural flexibility is found to be one of the determinants of amino acid modifications [61, 62].

Figure 5b shows the abundance of protein intrinsic disorder in the secondary interactome of beta-Catenin, which includes all the proteins that interact with the protein(s) in the first interactome of beta-Catenin. All the proteins in the secondary interactome were grouped by their interacting partners in the first interactome. In these groups, 23 have 10 or less partners in the secondary interactome, 15 have more than 10 but less than 100 partners, and only one protein (UBC) has about 500 interaction partners. Altogether, the total number of proteins in the secondary interactome is 1010. To show the abundance of intrinsic disorder, we used both the fraction of disordered residues and the total number of disordered residues because the interaction partners may have large variance in size. In fact, in the first interactome of beta-Catenin, two are short proteins (CTNNBIP1 and SKP1) of about 100 residues, four (APC, CREBBP, CDH8, and TRRAP) are large proteins with more than 2000 residues, and the rest 33 have from hundreds to a little more than 1000 residues. After using both measures, it can be seen that most of the interaction partners in the secondary interactome have either higher ratio of disordered residues or higher absolute number of disordered residues. Only in the last five groups on the x-axis (CDH1, RUVBL1, FBWX11, RUVBL2, and UBC), the proteins have smaller portions of interaction partners that have both high fraction and high absolute number of disordered residues. In conclusion, the majority of the proteins in the secondary interactome have significant amount of disordered residues.

### Disordered tails regulate the function of beta-Catenin

**Fig. 6:**
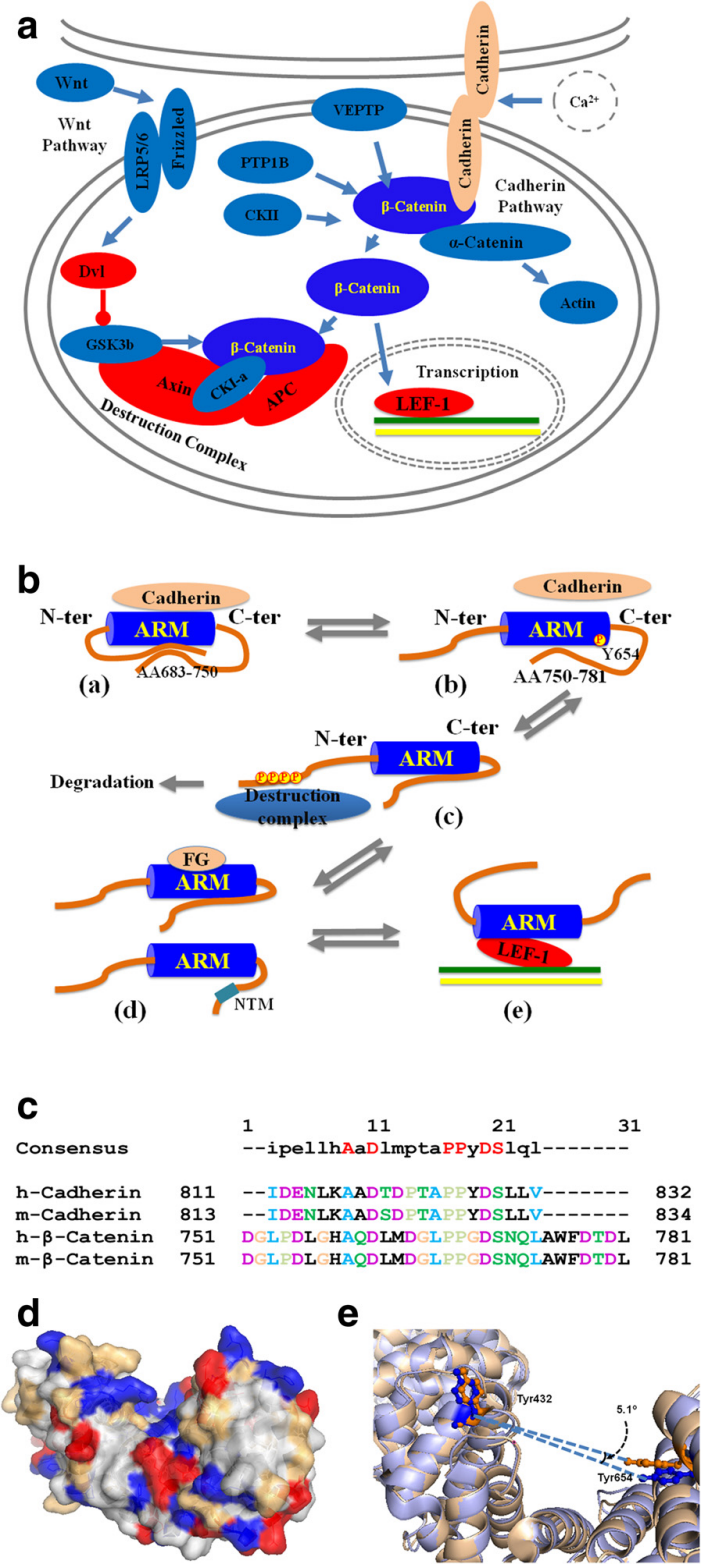
Disordered tails regulate the function of beta-Catenin in two pathways. (A) Key players in the cadherin junction formation pathway and the Wnt signaling pathway. Proteins in red color are intrinsically disordered, while proteins in blue color are basically structured. Beta-Catenin was shown in dark blue to be differentiated from other proteins. Cadherin was shown in apricot due to its large amount of intrinsically disordered residues. (B) Regulatory roles of disordered tails on the functions of beta-Catenin in both cadherin junction formation and Wnt signaling pathways. ARM is the armadillo domain of beta-Catenin. N-ter and C-ter stand for the N-terminal and C-terminal tails of beta-Catenin. (a) – (e) represent five different functional stages of beta-Catenin. “P” indicates phosphorylation. “FG” stands for phenylalanine-glycine(FG)-repeat containing proteins in the nuclear pore complex. “NTM” refers to nuclear translocation motif. The destruction complex is composed of CKI-alpha, GSK-3beta, Axin, and APC. (C) Conserved sequential patterns among mouse cadherin (UniPortKB Entry: P09803, AA813-834), human cadherin (UniPortKB Entry: P12830, AA811-832), mouse beta-Catenin (UniProtKB Entry: Q02248, AA751-781), and human beta-Catenin (UniPortKB Entry: P35222, AA751-781). (D) Pocket formed by AA400-663 (PDB id: 1JDN). Blue: positive charged residues; Red: negative charge residues; Gray: hydrophobic residues; Light yellow: other residues. (E) Change of the direction of the line formed between the oxygen atom on the side chain of Tyr654 and the Cα atom of Tyr432 in two PDB structures (Blue: PDB 2Z6H; Apricot: PDB 1QZ7). The angle associated with the change of the direction implies the structural variation of the binding pocket. The RMSD calculated from these two structures for the segment from AA576 to AA685 is 0.75 angstroms

Beta-catenin is predominantly involved in two pathways: the cadherin junction formation pathway and the Wnt signaling pathway. The major component molecules in these two pathways at different subcellular locations were presented in Fig. 6a. Many proteins associated with the Wnt signaling pathway have large fraction of disordered residues and therefore are labeled in red. Figure 6b describes the functional roles of disordered tails at five representative stages in both of the afore-mentioned pathways. In this figure, stages (a) and (b) correspond to the cadherin junction formation pathway. Stages (c), (d), and (e) are related to the Wnt signaling pathway.

In the cadherin junction formation pathway, cadherin interacts with ARM domain on a region spanning from the 12th to the 7th armadillo repeats [2]. This interaction is enhanced by the association between the N-terminal tail and the ARM domain of beta-Catenin [22]. A specific C-terminal segment AA683-AA750 also interacts with the N-terminal tail to facilitate the association between the N-terminal tail and the ARM domain [22]. Interestingly, a different C-terminal segment of beta-Catenin competes with cadherin on binding the ARM domain [22]. Figure 6c shows that a C-terminal segment on beta-Catenin starting at Leu-753 is highly conserved to the corresponding binding motif on cadherin. This newly identified location of the C-terminal segment is different from previously reported segment that spans from Gln-760 to the end of beta-Catenin [22]. Besides the competing interactions among different regions, the phosphorylation of Tyr-654 that is close to the C-terminal tail eliminates the interaction between cadherin and the ARM domain of beta-Catenin [22]. These complex regulatory roles of disordered tails of beta-Catenin are illustrated in (a) and (b) of Fig. 6b.

The contacting residues on the interface between human cadherin and human beta-Catenin include: Ile811, Leu815, Asp819, Pro826, Tyr827, Asp828, Ser829, and Leu831 on cadherin, and Ala656, Tyr654, Asn516, Arg474, His470.Tyr469, and Asn426 on beta-Catenin [2]. In which, Tyr-654 on beta-Catenin is critical for the association between cadherin and beta-Catenin. Its phosphorylation disrupts the interaction between cadherin and beta-Catenin [60, 63, 64]. The phosphorylation also decreases the affinity between the ARM domain and the C-terminal tail of beta-Catenin [60]. From a theoretical point of view, phosphorylation on Tyr-654 changes the local polarity of the ARM domain, leading to the interactional and functional modifications of the ARM domain. As shown in Fig. 6d, the interface composed of AA400-663 on the ARM domain is actually a pocket and is enriched of charged and hydrophobic residues, of which the combination is ideal for mediating protein-protein interaction. In addition, structural comparison of the ARM domains in two different structures, which are obtained before and after binding to a motif, shows that the RMSD value associated with the structural change between these two structures for the region from AA576 to AA685 is 0.75 angstroms. The direction of the line formed between the oxygen atom on the side chain of Tyr-654 and the Cα atom of Tyr432 is changed by 5.1° after the motif binding. These results may indicate that the structural variation caused by motif binding is negligible.

Once the interaction between beta-Catenin and its partners is eliminated, the concentration of free beta-Catenin in cytoplasma will be increased. The free beta-Catenin may enter one of two possible processes: (1) the N-terminal tail of free beta-Catenin undergoes phosphorylation and ubiquitination that lead to the degradation of beta-Catenin [1]; (2) free beta-Catenin transports into nucleus to activate gene expression. These two processes comprise the Wnt signaling pathway shown in (c), (d), and (e) of Fig. 6b. At stage (c), CKI-alpha phosphorylates Ser-45 first and then GSK-3beta phosphorylates Thr-41, Ser-37, and Ser-33 successively. Therefore, the N-terminal tail must be free from binding partners for the phosphorylation and subsequent ubiquitination modifications, but the C-terminal tail may or may not be bound to other molecules. The phosphorylation process is facilitated by Axin and APC, which are two large disordered proteins, through forming a destruction complex by binding beta-Catenin, CKI-alpha, and GSK-3beta. The disordered segments in Axin and APC bind to CKI-alpha, GSK-3beta, and the ARM domain of beta-Catenin. The presence of Axin and APC is assumed to increase the local concentration of both enzymes and substrate to accelerate the reaction [16].

When Wnt molecules are present, the destruction complex could not be formed. Therefore, beta-Catenin will accumulate in cytoplasma and transport from cytoplasma into nucleus as shown at stage (d). Different translocation mechanisms have been reported. The translocation can be either independent of the interaction with phenylalanine-glycine(FG)-repeats in the nuclear pore complex [30], or dependent on the interaction between the ARM domain of beta-Catenin and the FG repeats [17, 18]. FG repeats in nuclear pore complexes normally have 200 ~ 400 residues and are intrinsically disordered [65]. In the process of nuclear translocation, both the C-ter tail and the ARM domain of beta-Catenin are required [66]. Once being transported into nucleus, beta-Catenin will interact with TCF/LEF family proteins to trigger the transcription of target genes as shown at stage (e). Although only the ARM domain is required for the association between beta-Catenin and LEF-1 [19–21], both tails of beta-Catenin are observed to promote the transactivation of downstream genes [67, 68].

## Discussions

Beta-catenin has two major functions: regulating the formation of cadherin junction and processing Wnt signaling. These functions are performed synergistically by incorporating three sequential regions of beta-Catenin, including the N-terminal tail, the ARM domain, and the C-terminal tail. The ARM domain is composed of 12 helical segments and forms a structured domain, but both N- and C-terminal tails are disordered. In this study, sequential motifs, structural features, mutations, interaction partners, and modifications on both tails were integrated together to characterize the regulatory roles of the disordered tails. This is for the first time that all the five different aspects have been taken into consideration to characterize the regulatory roles of disordered tails of beta-Catenin.

### Synergy between ARM and tails

Beta-Catenin interacts with its partners using not only the ARM domain but also the tails. The ARM domain forms several binding pockets, while the tails contain multiple predicted binding motifs and ELM linear motifs. The ARM domain frequently binds to short flexible motifs of the partners, and the disordered tails interact with structured domains of the partners. Therefore, protein intrinsic disorder is very often involved in the interaction between beta-Catenin and its partners. Since the interactions involving disordered regions are often characterized by high-specificity and low-affinity [69], disordered motifs in the signaling pathways facilitate the regulation of the functions.

### Functional motifs and sites in tails

Binding motif predictors may be used not only for identifying binding sites, but also for inferring the mechanisms of different binding processes since different predictors are designed for different types of binding motifs. Three binding motif predictors, including MoRF, MoRFpred, and ANCHOR, were used in this study. ANCHOR is able to identify binding motifs composed by mainly hydrophobic residues. MoRF predicts motifs undergoing coil-to-helix transition upon binding to partners. MoRFpred identifies motifs associated with structural transition from coil to all three types of secondary structures. The correlation between binding motifs and secondary structures can be demonstrated by comparing the binding motifs to the results of secondary structure prediction from NetSurfP. As shown in Fig. 1, it is clear that all the predicted binding motifs in the N-terminal tail have helical structures, while the binding motifs at the C-terminal tail are not helices. The structural information of binding motifs can be further used to infer the structural requirements of binding events.

The heterogeneous distribution of the ELM motifs found in the N-terminal and C-terminal tails indicates different functional roles of the tails. All DEG motifs locate in the N-terminal tail. Therefore, the N-terminal tail is the only region responsible for the ubiquitination and degradation of beta-Catenin. Actually, the degradation process of beta-Catenin has been well characterized. The phosphorylation of serine and threonine residues on the N-terminal tail is the prerequisite of subsequent ubiquitination and degradation of beta-Catenin [23, 27]. The C-terminal tail has more DOC and MOD motifs than the N-terminal tail does. The coincident abundance of both DOC and MOD motifs in the C-terminal tail is expected and consistent, since the DOC motifs function in binding modifying enzymes, and the MOD motifs refer to modification sites. Therefore, the C-terminal tail may undergo many more modification events.

The C-terminal tail of beta-Catenin contains two neighboring segments that have contradictory functions. AA683-AA750 interacts with the N-terminal tail to facilitate the binding between the N-terminal tail and the ARM domain, and to further facilitate the interaction between beta-Catenin and cadherin. AA753-AA781, which is right after the previous motif, competing with cadherin on the interaction with the ARM domain. Since these two segments have well conserved sequential patterns and are enriched of functional motifs, it is expected that additional partners may be involved in the regulation of the interaction between these two motifs and the ARM domain.

Tyr-654 is a critical residue in regulating the interaction between the C-terminal tail and the ARM domain of beta-Catenin, as well as the interaction between beta-Catenin and cadherin. The spatial position of Tyr-654 is at the mouth of the binding pocket formed by the 7^th^ to the 12^th^ armadillo repeats. Therefore, when the pocket is occupied by a binding motif, the accessible surface area of Tyr-654 will be decreased significantly. By taking into consideration that the structural variation associated with motif binding is minor, it can be concluded that the phosphorylation of Tyr-654 is unlikely to occur when a binding motif is present, indicating the phosphorylation/dephosphorylation of Tyr-654 has to occur in the unbound state. Therefore, the pre-binding modification of Tyr-654 regulates the interaction between the ARM domain and its binding partners.

### Mutations on tails

High substitution rate is often observed in disordered proteins and regions. However, although both of the N- and C-terminal tails of beta-Catenin are disordered, they contain multiple conserved motifs (i.e., AA90 ~ AA130 in the N-terminal tail and AA774 ~ 781 in the C-terminal tail) and conserved sequential patterns (e.g., AA670 ~ AA680 and AA730 ~ AA740). These conserved regions actually overlap with predicted binding motifs and ELM linear motifs, indicating the conservation of functions. Even though, many segments with conserved amino acids or sequential patterns are still lack of functional annotation. AA90 ~ AA100 and AA140 ~ AA150 are two conserved motifs in the N-terminal tail. Both motifs are characterized as composites of hydrophobic residues and charged residues, with positive charged residues in the first motif and negative charged residues in the second motif. AA730 ~ AA740 and AA750 ~ AA760 are another two C-terminal segments with conserved sequential patterns involving hydrophobic, positively charged, and negatively charged residues on each of them.

Almost all the cancer-associated mutations on beta-Catenin are found in the N-terminal tail region, in which, over 70 % of the mutations locate in a short segment of 50 residues (AA25-AA75) at the N-terminal tail. This region accommodates Ser-45, Thr-41, Ser-37, and Ser-33, which are four critical residues in the Wnt signaling pathway. The phosphorylation of these four residues initiates the degradation of beta-Catenin. Therefore, it is conceivable that the mutations in this short region may eliminate the tight control on the degradation of beta-Catenin. Consequently, accumulated beta-Catenin turns on the expression of down-stream genes. In addition, the mutation in this short segment may also affect the function of beta-Catenin in the cadherin function formation pathway. In this pathway, the N-terminal tail of beta-Catenin interacts with the ARM domain to facilitate the interaction between beta-Catenin and cadherin. As shown in Figs. 1, 2b, and Additional file 1: Table S1, the N-terminal tail of beta-Catenin has three candidate binding motifs, locating at ~ AA25, ~AA80, and ~ AA130, respectively. The motif at ~ AA130 is sequentially next to the ARM domain. It is unlikely that this motif is able to “fold back” to interact with the ARM domain. As a result, only the motifs at ~ AA25 and ~ AA80 may perform the function of interacting with the ARM domain. Therefore, the afore-mentioned mutations in the region of AA25-AA75 may also abolish the interaction between the N-terminal tail and the ARM domain.

## Conclusions

Disordered tails of beta-Catenin interact with each other and the ARM domain to regulate the function of beta-Catenin synergistically. The tails contain many functional motifs. The types of motifs on both tails are significantly different, indicating divergence of their functional roles. Modifications on or near these motifs further modulate the function of the entire molecule. Mutations in tails are very often associated with diseases. Thorough characterization of the functional motifs in the tails helps further studies on the regulatory mechanisms of beta-Catenin.

## Abbreviations

APC, Adenomatous Polyposis Coli tumor repressor protein; ARM, Armadillo, Axin, Axis Inhibition Protein; CK1-alpha, Casein Kinase I alpha; CLV, CLeaVage site; DEG, DEGragon; DOC, modifying enzyme DOCking; ELM, Eukariotic Linear Motif; FG-repeats, phenylalanine-Glycine-repeats; GSK-3beta, Glycogen Synthase Kinase 3β; LIG, LIGand binding; MOD, post-translational MODification; MoRF, Molecular Recognition Feature; PP2A, Protein Phosphatase 2A; TRG, subcellular TaRGeting.

## Additional files

Additional file 1: Table S1. Eukaryotic linear motifs of beta-Catenin. (PDF 26 kb) Additional file 2: Table S2. List of beta-Catenin proteins from eight species. (PDF 8 kb) Additional file 3: Table S3. PDB structures of complexes formed between beta-Catenin and its partners. (PDF 13 kb) Additional file 4: Table S4. List of proteins in the first and secondary interactome of beta-Catenin. (PDF 198 kb)
