# Supplementary material for: Self-regulation of functional pathways by motifs inside the disordered tails of beta-catenin
Source: BMC Genomics. 2016 Aug 31;17(Suppl 5):484. doi: 10.1186/s12864-016-2825-9 (PMC5009561; doi:10.1186/s12864-016-2825-9)
Supplement: Additional file 1: Table S1. — Eukaryotic linear motifs of beta-Catenin. (PDF 26 kb) [file 12864_2016_2825_MOESM1_ESM.pdf]

Table S1. Eukaryotic Linear Motifs (ELMs) of beta-Catenin

Note	ELM name	Seq.	Start	END
	CLV_C14_Caspase3-7	SYLDS	29	33
	CLV_NRD_NRD_1	DRK	17	19
Confirmed	DEG_SCF_TRCP1_1	DSGIHS	32	37
	DEG_SCF_TRCP1_1	DSGIHS	32	37
	DOC_USP7_1	AAVSH	20	24
	DOC_USP7_1	AEPSQ	126	130
	LIG_Actin_WH2_2	HPTNVQRLA	118	135
	LIG_BIR_II_1	MATQA	1	5
	LIG_BRCT_BRCA1_1	FSQSF	70	74
	LIG_BRCT_BRCA1_1	PSTQF	110	114
	LIG_Clathr_ClatBox_1	LMELD	7	11
	LIG_EH1_1	KHAVVNLIN	133	141
	LIG_FHA_1	VDTSQVL	57	63
	LIG_FHA_2	MATQADL	1	7
	LIG_FHA_2	PETLDEG	100	106
	LIG_FHA_2	PSTQFDA	110	116
	LIG_SH2_SRC	YLDS	30	33
	LIG_SH2_STAT5	YLDS	30	33
	LIG_TRAF2_1	PEEE	52	55
	LIG_WD40_WDR5_VDV_2	TQADL	3	7
	LIG_WD40_WDR5_VDV_2	TTTAPSL	40	46
	LIG_WD40_WDR5_VDV_2	TQEQVADI	75	82
	LIG_WD40_WDR5_VDV_2	TRAQRVRA	89	96
	LIG_WD40_WDR5_VDV_2	YQDDAEL	142	148
	MOD_CK1_1	SGATTTA	37	43
	MOD_CK2_1	FPETLDE	99	105
	MOD_GlcNHglycan	DSGI	32	35
	MOD_GlcNHglycan	HSGA	36	39
	MOD_GlcNHglycan	LSGK	46	49
	MOD_GSK3_1	QQQSYLDS	26	33
Confirmed	MOD_GSK3_1	YLDSGIHS	30	37
	MOD_GSK3_1	YLDSGIHS	30	37
	MOD_GSK3_1	QGFSQSFT	68	75
	MOD_NEK2_1	FSQSFT	70	75
	MOD_PIKK_1	VDTSQVL	57	63
	MOD_PIKK_1	QGFSQSF	68	74
	MOD_PIKK_1	QSFTQEQ	72	78
	MOD_PIKK_1	IPSTQFD	109	115
	MOD_PIKK_1	AEPSQML	126	132
	TRG_PEX_1	WEQGF	66	70
	TRG_PEX_2	FSQSF	70	74
	CLV_NRD_NRD_1	RRT	549	551
	CLV_PCSK_KEX2_1	RRT	549	551
	DOC_CYCLIN_1	KLLHP	496	500
	DOC_PP2B_LxvP_1	LHPP	498	501
	DOC_USP7_1	PVDSV	247	251

	DOC_WW_Pin1_4	IMRSPQ	188	193
Confirmed	DOC_WW_Pin1_4	MLGSPV	243	248
	DOC_WW_Pin1_4	MLGSPV	243	248
	DOC_WW_Pin1_4	LLYSPI	602	607
	LIG_14-3-3_2	RGLNTIP	591	597
	LIG_14-3-3_3	RTYTYE	329	334
	LIG_eIF4E_1	YTYEKLL	331	337
	LIG_FHA_1	QNTNDVE	203	209
	LIG_FHA_1	LNTIPLF	593	599
	LIG_FHA_1	PLTELLH	639	645
	LIG_FHA_2	AATKQEG	391	397
	LIG_LIR_Gen_1	TYTYEKL	330	336
	LIG_LIR_Nem_3	TYTYEKL	330	336
	LIG_SH2_GRB2	YGNQ	306	309
	LIG_SH2_STAT3	YGNQ	306	309
	LIG_SH2_STAT5	YTYE	331	334
	LIG_SUMO_SIM_par_1	KVLSVCSS	345	352
	LIG_TRAF2_1	TKQE	393	396
	LIG_TYR_ITIM	LLYSPI	602	607
	LIG_WD40_WDR5_VDV_2	EAEGATA	632	638
	MOD_CK1_1	SVCSSNK	348	354
	MOD_CK1_1	SNLTCNN	425	431
	MOD_CK2_1	HQLSKKE	176	182
	MOD_CK2_1	DAATKQE	390	396
	MOD_CK2_1	LLYSPIE	602	608
	MOD_CK2_1	LLHSRNE	643	649
	MOD_GlcNHglycan	KSGG	233	236
	MOD_GlcNHglycan	ASGG	317	320
	MOD_GSK3_1	DVETARCT	207	214
	MOD_GSK3_1	MLGSPVDS	243	250
	MOD_GSK3_1	KVLSVCSS	345	352
	MOD_GSK3_1	RNLSDAAT	386	393
	MOD_GSK3_1	HQDTQRRT	544	551
	MOD_GSK3_1	EGATAPLT	634	641
	MOD_NEK2_1	LNKTNV	286	291
	MOD_NEK2_1	MGGTQQ	553	558
	MOD_NEK2_1	LLHSRN	643	648
	MOD_N-GLC_1	HNLSHH	219	224
	MOD_N-GLC_1	LNKTNV	286	291
	MOD_N-GLC_1	RNLSDA	386	391
	MOD_N-GLC_1	SNLTCN	425	430
	MOD_PIKK_1	TDPSQRL	371	377
	MOD_PIKK_1	HQDTQRR	544	550
	MOD_PIKK_1	MGGTQQQ	553	559
	MOD_PK_1	KVLSVCS	345	351
Confirmed	MOD_PKA_1	RRTSMGG	549	555
	MOD_PKA_1	RRTSMGG	549	555
	MOD_PKA_2	QRRTSMG	548	554
	MOD_ProDKin_1	MLGSPVD	243	249
	MOD_ProDKin_1	LLYSPIE	602	608

	TRG_ENDOCYTIC_2	YSPI	604	607
	TRG_NES_CRM1_1	EGMEGLLGI	396	412
	CLV_C14_Caspase3-7	DLMDG	761	765
	CLV_PCSK_KEX2_1	KRL	672	674
	CLV_PCSK_PC1ET2_1	KRL	672	674
	DOC_CYCLIN_1	KRLSV	672	676
	DOC_MAPK_1	KKRLSVEL	671	678
	DOC_USP7_1	PGDSN	768	772
	LIG_BIR_III_4	DALGM	727	731
	LIG_BRCT_BRCA1_1	TSSLF	679	683
	LIG_FHA_1	ELTSSLF	677	683
	LIG_FHA_1	NETADLG	691	697
	LIG_LIR_Nem_3	DPSYRSF	713	719
Confirmed	LIG_PDZ_Class_1	WFDTDL	776	781
	LIG_PDZ_Class_1	WFDTDL	776	781
	LIG_SH3_5	PGADY	744	748
	LIG_WD40_WDR5_VDV_1	DLGLDI	695	700
	LIG_WD40_WDR5_VDV_2	DYKKRLSV	669	676
	LIG_WD40_WDR5_VDV_2	SVEL	675	678
	LIG_WD40_WDR5_VDV_2	ETADL	692	696
	LIG_WD40_WDR5_VDV_2	DLGLDI	695	700
	LIG_WD40_WDR5_VDV_2	DGLPDL	751	756
	MOD_CK1_1	SYRSFHS	715	721
	MOD_GlcNHglycan	HSGG	720	723
	MOD_GSK3_1	KRLSVELT	672	679
	MOD_GSK3_1	LTSSLFRT	678	685
	MOD_NEK2_1	LTSSLF	678	683
	MOD_N-GLC_1	WNETAD	690	695
	MOD_PK_1	KRLSVEL	672	678
	MOD_PKA_1	KRLSVEL	672	678
Confirmed	MOD_PKA_2	KRLSVEL	672	678
	MOD_PKA_2	FRMSEDK	660	666
	MOD_PKA_2	KRLSVEL	672	678
	TRG_ENDOCYTIC_2	YRSF	716	719
