# Supplementary material for: Self-regulation of functional pathways by motifs inside the disordered tails of beta-catenin
Source: BMC Genomics. 2016 Aug 31;17(Suppl 5):484. doi: 10.1186/s12864-016-2825-9 (PMC5009561; doi:10.1186/s12864-016-2825-9)
Supplement: Additional file 2: Table S2. — List of beta-Catenin proteins from eight species. (PDF 8 kb) [file 12864_2016_2825_MOESM2_ESM.pdf]

Table S2. Beta-Catenin from eight species.

Species	UniProtKB Entry
Human	P35222
Mouse	Q02248
Bovine	Q0VCX4
Dog	B6V8E6
Turtle	Q5R2I4
Frog	Q63ZT4
Jellyfish	A4UAH1
Urchin	P35223
