# Supplementary material for: Self-regulation of functional pathways by motifs inside the disordered tails of beta-catenin
Source: BMC Genomics. 2016 Aug 31;17(Suppl 5):484. doi: 10.1186/s12864-016-2825-9 (PMC5009561; doi:10.1186/s12864-016-2825-9)
Supplement: Additional file 3: Table S3. — PDB structures of complexes formed between beta-Catenin and its partners. (PDF 13 kb) [file 12864_2016_2825_MOESM3_ESM.pdf]

**Table S3. PDB structures of complexes formed between beta-Catenin and its partners**

| PDB ID | beta-Catenin | beta-Catenin Conformation | Partner | Partner Conformation |
|---|---|---|---|---|
| 4ONS | N-ter AA84-143 | Helical segment | alpha-Catenin | Helical bundle |
| 4DJS | Arm | Arm Domain | MK8 | small ligand |
| 1DOW | N-ter AA118-149 | Helical segment | alpha-Catenin | Helical bundle |
| 2G57 | N-ter AA19-44 | Helical segment | Antibody | ~~~ |
| 1QZ7 | Arm | Arm Domain | Axin | Helical segment |
| 3OUW | Arm | Arm Domain | Lef-1 | Extended helical segment |
| 1G3J | Arm | Arm Domain | XTCF3-CBD | Extended helical segment |
| 1I7X | Arm | Arm Domain | E-cadherin | Extended helical segment |
| 1LUJ | Arm | Arm Domain | ICAT | Extended helical segment |
| 1JPW | Arm | Arm Domain | Tcf-4 | Extended helical segment |
| 3OUX | Arm | Arm Domain | Lef-1 | Extended helical segment |
| 3DIW | C-ter AA772-781 | beta-Strand | TIP-1 | Structured domain |
| 1T08 | Arm | Arm Domain | ICAT/APC | Helical/coiled motifs |
| 3SL9 | Arm | Arm Domain | Bcl9 | Helical segment |
| 1I7W | Arm | Arm Domain | E-cadherin | Extended helical segment |
| 1JDH | Arm | Arm Domain | HTCF-4 | Helical segment |
| 3TX7 | Arm | Arm Domain | LRH-1 | Structured domain |
| 1V18 | Arm | Arm Domain | APC | Extended helical segment |
| 1JPP | Arm | Arm Domain | APC | Helical segment |
| 1M1E | Arm | Arm Domain | ICAT | Helical segment |
| 1TH1 | Arm | Arm Domain | APC | Extended helical segment |
| 2GL7 | Arm | Arm Domain | BCL9/Tcf4 | Extended helical segment |
| 1P22 | N-ter AA30-40 | beta-Strand | TrCP1/Skp1 | Structured domain |
| 3C2G | Arm | Arm Domain | POP-1 | Coiled motif |
