# Supplementary material for: Self-regulation of functional pathways by motifs inside the disordered tails of beta-catenin
Source: BMC Genomics. 2016 Aug 31;17(Suppl 5):484. doi: 10.1186/s12864-016-2825-9 (PMC5009561; doi:10.1186/s12864-016-2825-9)
Supplement: Additional file 4: Table S4. — List of proteins in the first and secondary interactome of beta-Catenin. (PDF 198 kb) [file 12864_2016_2825_MOESM4_ESM.pdf]

Table S4. Proteins in the first and secondary interactomes of beta-Catenin

I. Genes/proteins in the first interactome of betaCatenin

Query Gene	UniProtKB Entry	Genes in the Interactome	UniProtKB Entry	Protein Name
CTNNB1	P35222	MUC1	P15941	Mucin-1
		BTRC	Q9Y297	F-box/WD repeat-containing protein 1A
		AR	A0A0B4J1T2	Androgen receptor
		SMAD3	P84022	Mothers against decapentaplegic homolog 3
		UBC	P0CG48	Polyubiquitin-C
		PSEN1	P49768	Presenilin-1
		NR5A2	O00482	Nuclear receptor subfamily 5 group A member 2
		TRRAP	Q9Y4A5	Transformation/transcription domain-associated protein
		AXIN1	O15169	Axin-1
		HDAC1	Q13547	Histone deacetylase 1
		GSK3B	P49841	Glycogen synthase kinase-3 beta
		TCF4	P15884	Transcription factor 4
		CUL1	Q13616	Cullin-1
		RUVBL1	Q9Y265	RuvB-like 1
		CTNNA1	P35221	Catenin alpha-1
		CHD8	Q9HCK8	Chromodomain-helicase-DNA-binding protein 8
		PTPRF	P10586	Receptor-type tyrosine-protein phosphatase F
		PARD3	Q8TEW0	Partitioning defective 3 homolog
		AXIN2	Q9Y2T1	Axin-2
		EGFR	P00533	Epidermal growth factor receptor
		MEN1	O00255	Menin
		TCF7L2	Q9NQB0	Transcription factor 7-like 2
		FBXW11	Q9UKB1	F-box/WD repeat-containing protein 11

		CTNNBIP1	Q9NSA3	Beta-catenin-interacting protein 1
		CDH5	P33151	Cadherin-5
		APC	P25054	Adenomatous polyposis coli protein
		SKP1	P63208	S-phase kinase-associated protein 1
		CREBBP	Q92793	CREB-binding protein
		CDH1	P12830	Cadherin-1
		RUVBL2	X6R2L4	RuvB-like 2
		ERBB2	P04626	Receptor tyrosine-protein kinase erbB-2
		LEF1	Q9UJU2	Lymphoid enhancer-binding factor 1
		BCL9	O00512	B-cell CLL/lymphoma 9 protein
		CDH3	P22223	Cadherin-3
		TCF7	P36402	Transcription factor 7
		CDH2	P19022	Cadherin-2
		PITX2	Q99697	Pituitary homeobox 2
		RBBP5	Q15291	Retinoblastoma-binding protein 5
		APC2	O95996	Adenomatous polyposis coli protein 2

II. Genes/proteins in the Secondary Interactome of beta-Catenin (Genes interacting with the genes in the first interactome of beta-Catenin)

Query Gene	UniProtKB Entry	Genes in the Interactome	UniProtKB Entry	Protein Name
MUC1	P15941	SRC	P12931	Proto-oncogene tyrosine-protein kinase Src
		EGFR	P00533	Epidermal growth factor receptor
		CTNNB1	P35222	Catenin beta-1
BTRC	Q9Y297	OGT	O15294	UDP-N-acetylglucosamine--peptide N-acetylglucosaminyltransferase 110 kDa subunit
		HIVEP1	P15822	Zinc finger protein 40
		C13orf34	B5LMG6	Aurora borealis
		NUDC	Q9Y266	Nuclear migration protein nudC
		NFKB2	Q00653	Nuclear factor NF-kappa-B p100 subunit

		SKP1	P63208	S-phase kinase-associated protein 1
		USP47	Q96K76	Ubiquitin carboxyl-terminal hydrolase 47
		IKBKB	J3KNS5	Inhibitor of nuclear factor kappa-B kinase subunit beta
		NFKB1	P19838	Nuclear factor NF-kappa-B p105 subunit
		APC	P25054	Adenomatous polyposis coli protein
		HIVEP2	P31629	Transcription factor HIVEP2
		CTNNB1	P35222	Catenin beta-1
		CDC25A	P30304	M-phase inducer phosphatase 1
		ATF4	P18848	Cyclic AMP-dependent transcription factor ATF-4
		MED20	Q9H944	Mediator of RNA polymerase II transcription subunit 20
		CLPX	O76031	ATP-dependent Clp protease ATP-binding subunit clpX-like, mitochondrial
		SMAD4	Q13485	Mothers against decapentaplegic homolog 4
		CLSPN	Q9HAW4	Claspin
		SNAI1	O95863	Zinc finger protein SNAI1
		TUBGCP3	Q96CW5	Gamma-tubulin complex component 3
		CDC34	P49427	Ubiquitin-conjugating enzyme E2 R1
		NRD1	B1AKJ5	Nardilysin
		FBXW11	Q9UKB1	F-box/WD repeat-containing protein 11
		PDCD4	Q53EL6	Programmed cell death protein 4
		NFKBIA	P25963	NF-kappa-B inhibitor alpha
		CUL1	Q13616	Cullin-1
AR	A0A0B4J1T2	TGFB1I1	O43294	Transforming growth factor beta-1-induced transcript 1 protein
		PIAS2	A0A0A0MS92	E3 SUMO-protein ligase PIAS2
		NCOA4	Q13772	Nuclear receptor coactivator 4
		NCOA2	Q15596	Nuclear receptor coactivator 2
		MDM2	J3KN53	E3 ubiquitin-protein ligase Mdm2
		RB1	P06400	Retinoblastoma-associated protein
		EGFR	P00533	Epidermal growth factor receptor
		KLK3	P07288	Prostate-specific antigen
		RNF14	Q9UBS8	E3 ubiquitin-protein ligase RNF14

		RCHY1	Q96PM5	RING finger and CHY zinc finger domain-containing protein 1
		EP300	Q09472	Histone acetyltransferase p300
		SRC	P12931	Proto-oncogene tyrosine-protein kinase Src
		STUB1	Q9UNE7	E3 ubiquitin-protein ligase CHIP
		HDAC1	Q13547	Histone deacetylase 1
		TMPRSS2	O15393	Transmembrane protease serine 2
		SMAD3	P84022	Mothers against decapentaplegic homolog 3
		RNF4	P78317	E3 ubiquitin-protein ligase RNF4
		SMARCA4	P51532	Transcription activator BRG1
		UBC	P0CG48	Polyubiquitin-C
		HSP90AA1	P07900	Heat shock protein HSP 90-alpha
		CREBBP	Q92793	CREB-binding protein
		CTNNB1	P35222	Catenin beta-1
		PA2G4	Q9UQ80	Proliferation-associated protein 2G4
		SP1	P08047	Transcription factor Sp1
		KAT5	Q92993	Histone acetyltransferase KAT5
		NCOA3	Q9Y6Q9	Nuclear receptor coactivator 3
		NCOA1	Q15788	Nuclear receptor coactivator 1
		NCOR2	Q9Y618	Nuclear receptor corepressor 2
		NCOR1	O75376	Nuclear receptor corepressor 1
		STAT3	P40763	Signal transducer and activator of transcription 3
SMAD3	P84022	AR	A0A0B4J1T2	Androgen receptor
		CTNNB1	P35222	Catenin beta-1
		HDAC1	Q13547	Histone deacetylase 1
		JUN	P05412	Transcription factor AP-1
		NEDD9	Q14511	Enhancer of filamentation 1
		RUNX2	Q13950	Runt-related transcription factor 2
		SKI	P12755	Ski oncogene
		MAPK1	P28482	Mitogen-activated protein kinase 1
		SMURF2	Q9HAU4	E3 ubiquitin-protein ligase SMURF2

		KAT2B	Q92831	Histone acetyltransferase KAT2B
		ZEB2	O60315	Zinc finger E-box-binding homeobox 2
		SMAD2	Q15796	Mothers against decapentaplegic homolog 2
		CREBBP	Q92793	CREB-binding protein
		TFE3	P19532	Transcription factor E3
		SP1	P08047	Transcription factor Sp1
		PIN1	Q13526	Peptidyl-prolyl cis-trans isomerase NIMA-interacting 1
		SKIL	P12757	Ski-like protein
		EP300	Q09472	Histone acetyltransferase p300
		ZFYVE9	O95405	Zinc finger FYVE domain-containing protein 9
		RNF111	Q6ZNA4	E3 ubiquitin-protein ligase Arkadia
		SMAD4	Q13485	Mothers against decapentaplegic homolog 4
		SMURF1	Q9HCE7	E3 ubiquitin-protein ligase SMURF1
		TRIM33	Q9UPN9	E3 ubiquitin-protein ligase TRIM33
		UBC	P0CG48	Polyubiquitin-C
UBC	P0CG48	ABCA1	O95477	ATP-binding cassette sub-family A member 1
		ACTB	P60709	Actin, cytoplasmic 1
		APOBEC3G	Q9HC16	DNA dC->dU-editing enzyme APOBEC-3G
		AR	A0A0B4J1T2	Androgen receptor
		ARHGEF7	Q14155	Rho guanine nucleotide exchange factor 7
		ATP1B1	P05026	Sodium/potassium-transporting ATPase subunit beta-1
		ATXN3	D3VVP3	Ataxin 3 variant h
		AUP1	Q9Y679	Ancient ubiquitous protein 1
		BECN1	Q14457	Beclin-1
		BIRC6	Q9NR09	Baculoviral IAP repeat-containing protein 6
		BMI1	P35226	Polycomb complex protein BMI-1
		BRCA1	P38398	Breast cancer type 1 susceptibility protein
		BRCA2	P51587	Breast cancer type 2 susceptibility protein
		BRCC3	H9KVA9	Lys-63-specific deubiquitinase BRCC36
		CCDC50	Q8IVM0	Coiled-coil domain-containing protein 50

CCT4	P50991	T-complex protein 1 subunit delta
CDK9	P50750	Cyclin-dependent kinase 9
CDKN2A	A0A0A0MRI0	Cyclin-dependent kinase inhibitor 2A
COPS5	Q92905	COP9 signalosome complex subunit 5
CRKL	P46109	Crk-like protein
CXCR4	P61073	C-X-C chemokine receptor type 4
DAPK1	P53355	Death-associated protein kinase 1
DDX58	O95786	Probable ATP-dependent RNA helicase DDX58
DHX9	Q08211	ATP-dependent RNA helicase A
DNTT	P04053	DNA nucleotidylexotransferase
E2F1	Q01094	Transcription factor E2F1
EIF3A	Q14152	Eukaryotic translation initiation factor 3 subunit A
EPN1	Q9Y6I3	Epsin-1
EPS15	P42566	Epidermal growth factor receptor substrate 15
ETS1	P14921	Protein C-ets-1
FAF1	Q9UNN5	FAS-associated factor 1
FAM175A	Q6UWZ7	BRCA1-A complex subunit Abraxas
FBP1	P09467	Fructose-1,6-bisphosphatase 1
FGFR1	P11362	Fibroblast growth factor receptor 1
FOXO1	Q12778	Forkhead box protein O1
FOXO4	P98177	Forkhead box protein O4
FTL	P02792	Ferritin light chain
FYN	P06241	Tyrosine-protein kinase Fyn
G6PD	P11413	Glucose-6-phosphate 1-dehydrogenase
GNAS	Q5JWF2	Guanine nucleotide-binding protein G(s) subunit alpha isoforms XLas
GPI	P06744	Glucose-6-phosphate isomerase
H2AFX	P16104	Histone H2AX
HDAC1	Q13547	Histone deacetylase 1
HIST2H2BE	Q16778	Histone H2B type 2-E
HNRNPA3	P51991	Heterogeneous nuclear ribonucleoprotein A3

HNRNPH1	P31943	Heterogeneous nuclear ribonucleoprotein H
HNRNPK	P61978	Heterogeneous nuclear ribonucleoprotein K
HSF2	Q03933	Heat shock factor protein 2
HTT	P42858	Huntingtin
IGHG1	A0A0A0MS08	Ig gamma-1 chain C region (Fragment)
IKBKE	Q14164	Inhibitor of nuclear factor kappa-B kinase subunit epsilon
IKBKG	Q9Y6K9	NF-kappa-B essential modulator
INPP5D	Q92835	Phosphatidylinositol 3,4,5-trisphosphate 5-phosphatase 1
IRAK1	P51617	Interleukin-1 receptor-associated kinase 1
ITCH	Q96J02	E3 ubiquitin-protein ligase Itchy homolog
JAK2	O60674	Tyrosine-protein kinase JAK2
JUN	P05412	Transcription factor AP-1
KLF5	Q13887	Krueppel-like factor 5
KRT18	P05783	Keratin, type I cytoskeletal 18
L1CAM	P32004	Neural cell adhesion molecule L1
LDHA	P00338	L-lactate dehydrogenase A chain
MAP3K1	Q13233	Mitogen-activated protein kinase kinase kinase 1
MAP3K5	Q99683	Mitogen-activated protein kinase kinase kinase 5
MAP3K7	O43318	Mitogen-activated protein kinase kinase kinase 7
MAVS	Q7Z434	Mitochondrial antiviral-signaling protein
MCL1	Q07820	Induced myeloid leukemia cell differentiation protein Mcl-1
MDC1	Q14676	Mediator of DNA damage checkpoint protein 1
MDM2	J3KN53	E3 ubiquitin-protein ligase Mdm2
MDM4	O15151	Protein Mdm4
MYC	P01106	Myc proto-oncogene protein
MYLIP	Q8WY64	E3 ubiquitin-protein ligase MYLIP
NCOA3	Q9Y6Q9	Nuclear receptor coactivator 3
NEDD4	P46934	E3 ubiquitin-protein ligase NEDD4
NEDD4L	Q96PU5	E3 ubiquitin-protein ligase NEDD4-like
NFE2L1	J9JIE5	Nuclear factor erythroid 2-related factor 1

NFE2L2	Q16236	Nuclear factor erythroid 2-related factor 2
NHLRC1	Q6VVB1	E3 ubiquitin-protein ligase NHLRC1
NTRK1	J3KP20	Tyrosine-protein kinase receptor
OCLN	Q16625	Occludin
OTUD7B	Q6GQQ9	OTU domain-containing protein 7B
PARK2	O60260	E3 ubiquitin-protein ligase parkin
PARP1	P09874	Poly [ADP-ribose] polymerase 1
PCNA	P12004	Proliferating cell nuclear antigen
PELI1	Q96FA3	E3 ubiquitin-protein ligase pellino homolog 1
PFKFB3	Q16875	6-phosphofructo-2-kinase/fructose-2,6-bisphosphatase 3
PHGDH	O43175	D-3-phosphoglycerate dehydrogenase
PIAS2	A0A0A0MS92	E3 SUMO-protein ligase PIAS2
PIAS3	Q9Y6X2	E3 SUMO-protein ligase PIAS3
PJA1	Q8NG27	E3 ubiquitin-protein ligase Praja-1
PJA2	O43164	E3 ubiquitin-protein ligase Praja-2
PLAA	Q9Y263	Phospholipase A-2-activating protein
POLH	Q9Y253	DNA polymerase eta
POU5F1	Q01860	POU domain, class 5, transcription factor 1
PPIA	P62937	Peptidyl-prolyl cis-trans isomerase A
PPP2CA	P67775	Serine/threonine-protein phosphatase 2A catalytic subunit alpha isoform
PSMA7	O14818	Proteasome subunit alpha type-7
PSMC6	A0A087X2I1	26S protease regulatory subunit 10B
PSMD4	P55036	26S proteasome non-ATPase regulatory subunit 4
PTEN	P60484	Phosphatidylinositol 3,4,5-trisphosphate 3-phosphatase and dual-specificity protein phosphatase PTEN
PTTG1	O95997	Securin
RAC1	P63000	Ras-related C3 botulinum toxin substrate 1
RAD23B	P54727	UV excision repair protein RAD23 homolog B
RAN	J3KQE5	GTP-binding nuclear protein Ran (Fragment)
RASSF1	Q9NS23	Ras association domain-containing protein 1

RBCK1	Q9BYM8	RanBP-type and C3HC4-type zinc finger-containing protein 1
RELA	Q04206	Transcription factor p65
RFWD2	Q8NHY2	E3 ubiquitin-protein ligase RFWD2
RHOA	P61586	Transforming protein RhoA
RNF115	Q9Y4L5	E3 ubiquitin-protein ligase RNF115
RNF2	Q99496	E3 ubiquitin-protein ligase RING2
RNF5	Q99942	E3 ubiquitin-protein ligase RNF5
RNF8	O76064	E3 ubiquitin-protein ligase RNF8
RPL24	P83731	60S ribosomal protein L24
RPL7A	P62424	60S ribosomal protein L7a
RPS8	P62241	40S ribosomal protein S8
RUNX2	Q13950	Runt-related transcription factor 2
S100A10	P60903	Protein S100-A10
SCD	O00767	Acyl-CoA desaturase
SCNN1B	P51168	Amiloride-sensitive sodium channel subunit beta
SFPQ	P23246	Splicing factor, proline- and glutamine-rich
SGK1	O00141	Serine/threonine-protein kinase Sgk1
SH3KBP1	Q96B97	SH3 domain-containing kinase-binding protein 1
SIAH1	Q8IUQ4	E3 ubiquitin-protein ligase SIAH1
SKI	P12755	Ski oncogene
SMN1	Q16637	Survival motor neuron protein
SMURF1	Q9HCE7	E3 ubiquitin-protein ligase SMURF1
SPG20	Q8N0X7	Spartin
SPRY2	O43597	Protein sprouty homolog 2
SQSTM1	Q13501	Sequestosome-1
SRC	P12931	Proto-oncogene tyrosine-protein kinase Src
SREBF1	P36956	Sterol regulatory element-binding protein 1
STAM	Q92783	Signal transducing adapter molecule 1
STAT1	P42224	Signal transducer and activator of transcription 1-alpha/beta
SUMO1	P63165	Small ubiquitin-related modifier 1

SYK	P43405	Tyrosine-protein kinase SYK
SYVN1	Q86TM6	E3 ubiquitin-protein ligase synoviolin
TAX1BP1	Q86VP1	Tax1-binding protein 1
TFAP2A	P05549	Transcription factor AP-2-alpha
TGFBR1	P36897	TGF-beta receptor type-1
TGM2	P21980	Protein-glutamine gamma-glutamyltransferase 2
TKT	P29401	Transketolase
TNFRSF1B	P20333	Tumor necrosis factor receptor superfamily member 1B
TNK2	Q07912	Activated CDC42 kinase 1
TOM1	O60784	Target of Myb protein 1
TOP2A	P11388	DNA topoisomerase 2-alpha
TP73	O15350	Tumor protein p73
TRIM32	Q13049	E3 ubiquitin-protein ligase TRIM32
TUBB	P07437	Tubulin beta chain
TUBB2A	Q13885	Tubulin beta-2A chain
SSR4	P51571	Translocon-associated protein subunit delta
RPN1	P04843	Dolichyl-diphosphooligosaccharide--protein glycosyltransferase subunit 1
ADRM1	Q16186	Proteasomal ubiquitin receptor ADRM1
ACTG1	P63261	Actin, cytoplasmic 2
RNF185	Q96GF1	E3 ubiquitin-protein ligase RNF185
BIRC2	Q13490	Baculoviral IAP repeat-containing protein 2
MALT1	Q9UDY8	Mucosa-associated lymphoid tissue lymphoma translocation protein 1
HSPA6	P17066	Heat shock 70 kDa protein 6
UBASH3A	P57075	Ubiquitin-associated and SH3 domain-containing protein A
ARF6	P62330	ADP-ribosylation factor 6
HIST1H2AA	Q96QV6	Histone H2A type 1-A
CFLAR	O15519	CASP8 and FADD-like apoptosis regulator
UBR5	O95071	E3 ubiquitin-protein ligase UBR5
AURKB	Q96GD4	Aurora kinase B
DVL3	Q92997	Segment polarity protein dishevelled homolog DVL-3

HIST1H1C	P16403	Histone H1.2
ITGA5	P08648	Integrin alpha-5
RAD18	Q9NS91	E3 ubiquitin-protein ligase RAD18
EPS15L1	Q9UBC2	Epidermal growth factor receptor substrate 15-like 1
PDCD4	Q53EL6	Programmed cell death protein 4
FOXO3	O43524	Forkhead box protein O3
GCM1	Q9NP62	Chorion-specific transcription factor GCMa
BIRC3	Q13489	Baculoviral IAP repeat-containing protein 3
ALDOA	P04075	Fructose-bisphosphate aldolase A
HDAC6	Q9UBN7	Histone deacetylase 6
GBA	P04062	Glucosylceramidase
TUBG1	P23258	Tubulin gamma-1 chain
SF3B3	Q15393	Splicing factor 3B subunit 3
EGFR	P00533	Epidermal growth factor receptor
PPP2R1A	P30153	Serine/threonine-protein phosphatase 2A 65 kDa regulatory subunit A alpha isoform
CCNA2	P20248	Cyclin-A2
APOB	P04114	Apolipoprotein B-100
SMAD7	O15105	Mothers against decapentaplegic homolog 7
UBE2B	P63146	Ubiquitin-conjugating enzyme E2 B
TERF2	Q15554	Telomeric repeat-binding factor 2
RPS2	P15880	40S ribosomal protein S2
HSP90AB1	P08238	Heat shock protein HSP 90-beta
BRE	Q9NXR7	BRCA1-A complex subunit BRE
TUBA1C	Q9BQE3	Tubulin alpha-1C chain
BCL2L1	Q07817	Bcl-2-like protein 1
SNAI1	O95863	Zinc finger protein SNAI1
USP13	Q92995	Ubiquitin carboxyl-terminal hydrolase 13
KIT	P10721	Mast/stem cell growth factor receptor Kit
PSMC2	P35998	26S protease regulatory subunit 7

JAG1	P78504	Protein jagged-1
LCK	P06239	Tyrosine-protein kinase Lck
CNTN2	Q02246	Contactin-2
TUFM	P49411	Elongation factor Tu, mitochondrial
VAV1	A0A0A0MR07	Proto-oncogene vav
TAL1	P17542	T-cell acute lymphocytic leukemia protein 1
DENR	O43583	Density-regulated protein
ACO1	P21399	Cytoplasmic aconitate hydratase
RPL15	P61313	60S ribosomal protein L15
RNF11	Q9Y3C5	RING finger protein 11
NONO	Q15233	Non-POU domain-containing octamer-binding protein
KCTD10	Q9H3F6	BTB/POZ domain-containing adapter for CUL3-mediated RhoA degradation protein 3
CUL1	Q13616	Cullin-1
PRDX1	Q06830	Peroxiredoxin-1
EIF3F	O00303	Eukaryotic translation initiation factor 3 subunit F
IFNAR1	P17181	Interferon alpha/beta receptor 1
TRIM28	Q13263	Transcription intermediary factor 1-beta
HERPUD1	Q15011	Homocysteine-responsive endoplasmic reticulum-resident ubiquitin-like domain member 1 protein
UBA1	P22314	Ubiquitin-like modifier-activating enzyme 1
OPTN	Q96CV9	Optineurin
NEIL2	Q969S2	Endonuclease 8-like 2
USP8	P40818	Ubiquitin carboxyl-terminal hydrolase 8
SMAD4	Q13485	Mothers against decapentaplegic homolog 4
NFKB2	Q00653	Nuclear factor NF-kappa-B p100 subunit
TK1	P04183	Thymidine kinase, cytosolic
RNF168	Q8IYW5	E3 ubiquitin-protein ligase RNF168
FOS	P01100	Proto-oncogene c-Fos
IKBKB	J3KNS5	Inhibitor of nuclear factor kappa-B kinase subunit beta

IRS1	P35568	Insulin receptor substrate 1
DVL2	O14641	Segment polarity protein dishevelled homolog DVL-2
PSMD2	Q13200	26S proteasome non-ATPase regulatory subunit 2
SCN5A	Q14524	Sodium channel protein type 5 subunit alpha
IGF1R	P08069	Insulin-like growth factor 1 receptor
DDB2	Q92466	DNA damage-binding protein 2
KEAP1	Q14145	Kelch-like ECH-associated protein 1
TMEM173	Q86WV6	Stimulator of interferon genes protein
SP1	P08047	Transcription factor Sp1
NR3C1	P04150	Glucocorticoid receptor
FLT3	P36888	Receptor-type tyrosine-protein kinase FLT3
INSIG1	O15503	Insulin-induced gene 1 protein
CTNNB1	P35222	Catenin beta-1
CDC25A	P30304	M-phase inducer phosphatase 1
TARDBP	Q13148	TAR DNA-binding protein 43
SNCA	P37840	Alpha-synuclein
HNRNPL	P14866	Heterogeneous nuclear ribonucleoprotein L
TAB2	Q9NYJ8	TGF-beta-activated kinase 1 and MAP3K7-binding protein 2
CD86	P42081	T-lymphocyte activation antigen CD86
AIFM1	O95831	Apoptosis-inducing factor 1, mitochondrial
PSMD3	O43242	26S proteasome non-ATPase regulatory subunit 3
USP2	O75604	Ubiquitin carboxyl-terminal hydrolase 2
DIABLO	Q9NR28	Diablo homolog, mitochondrial
RIPK1	Q13546	Receptor-interacting serine/threonine-protein kinase 1
HSP90AA1	P07900	Heat shock protein HSP 90-alpha
SOD1	P00441	Superoxide dismutase [Cu-Zn]
TNFAIP3	P21580	Tumor necrosis factor alpha-induced protein 3
DDIT4	Q9NX09	DNA damage-inducible transcript 4 protein
TUBA1A	Q71U36	Tubulin alpha-1A chain
UBE2E1	P51965	Ubiquitin-conjugating enzyme E2 E1

CHFR	A0A096P6K8	E3 ubiquitin-protein ligase CHFR
RUNX3	Q13761	Runt-related transcription factor 3
KAT2A	Q92830	Histone acetyltransferase KAT2A
PDGFRB	P09619	Platelet-derived growth factor receptor beta
HSPA8	P11142	Heat shock cognate 71 kDa protein
NPM1	P06748	Nucleophosmin
ZFAND5	O76080	AN1-type zinc finger protein 5
SMAD3	P84022	Mothers against decapentaplegic homolog 3
TTYH2	Q9BSA4	Protein tweety homolog 2
CHEK1	J3KN87	Serine/threonine-protein kinase Chk1
PHB	P35232	Prohibitin
UBE2O	Q9C0C9	E2/E3 hybrid ubiquitin-protein ligase UBE2O
TANK	Q92844	TRAF family member-associated NF-kappa-B activator
IRF3	Q14653	Interferon regulatory factor 3
CBL	P22681	E3 ubiquitin-protein ligase CBL
IMPDH2	P12268	Inosine-5'-monophosphate dehydrogenase 2
MIB1	Q86YT6	E3 ubiquitin-protein ligase MIB1
NOS2	P35228	Nitric oxide synthase, inducible
IRF7	Q92985	Interferon regulatory factor 7
SMAD2	Q15796	Mothers against decapentaplegic homolog 2
PPARGC1A	Q9UBK2	Peroxisome proliferator-activated receptor gamma coactivator 1-alpha
CTBP1	Q13363	C-terminal-binding protein 1
TERF1	P54274	Telomeric repeat-binding factor 1
ERBB3	P21860	Receptor tyrosine-protein kinase erbB-3
HNRNPU	Q00839	Heterogeneous nuclear ribonucleoprotein U
TSC2	P49815	Tuberin
BCL2	P10415	Apoptosis regulator Bcl-2
CCT2	P78371	T-complex protein 1 subunit beta
TRIM21	P19474	E3 ubiquitin-protein ligase TRIM21
TAB3	A0A0A0MQY2	TGF-beta-activated kinase 1 and MAP3K7-binding protein 3 (Fragment)

USP25	Q9UHP3	Ubiquitin carboxyl-terminal hydrolase 25
AURKA	O14965	Aurora kinase A
NPLOC4	Q8TAT6	Nuclear protein localization protein 4 homolog
NOS1	P29475	Nitric oxide synthase, brain
USP16	Q9Y5T5	Ubiquitin carboxyl-terminal hydrolase 16
RCHY1	Q96PM5	RING finger and CHY zinc finger domain-containing protein 1
JUNB	P17275	Transcription factor jun-B
TSC1	Q92574	Hamartin
RUNX1	Q01196	Runt-related transcription factor 1
MAPK1	P28482	Mitogen-activated protein kinase 1
USP14	P54578	Ubiquitin carboxyl-terminal hydrolase 14
PSMD8	P48556	26S proteasome non-ATPase regulatory subunit 8
RPS3	P23396	40S ribosomal protein S3
DAXX	Q9UER7	Death domain-associated protein 6
ASB2	Q96Q27	Ankyrin repeat and SOCS box protein 2
STUB1	Q9UNE7	E3 ubiquitin-protein ligase CHIP
SENP3	Q9H4L4	Sentrin-specific protease 3
USP5	P45974	Ubiquitin carboxyl-terminal hydrolase 5
CDH1	P12830	Cadherin-1
MAPT	P10636	Microtubule-associated protein tau
HIF1A	Q16665	Hypoxia-inducible factor 1-alpha
RPLP0	P05388	60S acidic ribosomal protein P0
RNF114	Q9Y508	E3 ubiquitin-protein ligase RNF114
MET	P08581	Hepatocyte growth factor receptor
PRPF8	Q6P2Q9	Pre-mRNA-processing-splicing factor 8
GJA1	P17302	Gap junction alpha-1 protein
EEF2	P13639	Elongation factor 2
CDKN1A	P38936	Cyclin-dependent kinase inhibitor 1
RNF139	Q8WU17	E3 ubiquitin-protein ligase RNF139
GAPDH	P04406	Glyceraldehyde-3-phosphate dehydrogenase

PLCG1	P19174	1-phosphatidylinositol 4,5-bisphosphate phosphodiesterase gamma-1
LRP6	O75581	Low-density lipoprotein receptor-related protein 6
UBE2K	P61086	Ubiquitin-conjugating enzyme E2 K
UBE2N	P61088	Ubiquitin-conjugating enzyme E2 N
ABCB1	P08183	Multidrug resistance protein 1
DHX15	O43143	Pre-mRNA-splicing factor ATP-dependent RNA helicase DHX15
EIF4A1	P60842	Eukaryotic initiation factor 4A-I
LDLR	J3KMZ9	Low-density lipoprotein receptor (Fragment)
SLC40A1	Q9NP59	Solute carrier family 40 member 1
EIF4E	P06730	Eukaryotic translation initiation factor 4E
AMFR	Q9UKV5	E3 ubiquitin-protein ligase AMFR
CCNB1	P14635	G2/mitotic-specific cyclin-B1
RAD23A	P54725	UV excision repair protein RAD23 homolog A
VHL	P40337	Von Hippel-Lindau disease tumor suppressor
TERT	O14746	Telomerase reverse transcriptase
TUT1	J3KN81	Speckle targeted PIP5K1A-regulated poly(A) polymerase (Fragment)
OTUD5	Q96G74	OTU domain-containing protein 5
RABGEF1	Q9UJ41	Rab5 GDP/GTP exchange factor
PSMC5	P62195	26S protease regulatory subunit 8
FBL	P22087	rRNA 2'-O-methyltransferase fibrillarin
SKP2	Q13309	S-phase kinase-associated protein 2
DNM1L	O00429	Dynamin-1-like protein
SF3B1	O75533	Splicing factor 3B subunit 1
STAMBP	O95630	STAM-binding protein
CCNE1	P24864	G1/S-specific cyclin-E1
PRKCA	J3KN97	Protein kinase C alpha type (Fragment)
CLU	P10909	Clusterin
TP53	P04637	Cellular tumor antigen p53
SMURF2	Q9HAU4	E3 ubiquitin-protein ligase SMURF2
CDC34	P49427	Ubiquitin-conjugating enzyme E2 R1

ENO1	P06733	Alpha-enolase
KDR	P35968	Vascular endothelial growth factor receptor 2
ANXA1	P04083	Annexin A1
CFTR	P13569	Cystic fibrosis transmembrane conductance regulator
APC	P25054	Adenomatous polyposis coli protein
MAP1B	P46821	Microtubule-associated protein 1B
NOTCH3	Q9UM47	Neurogenic locus notch homolog protein 3
RPL18	J3QQ67	60S ribosomal protein L18 (Fragment)
IREB2	A0A0A6YY96	Iron-responsive element-binding protein 2
BARD1	Q99728	BRCA1-associated RING domain protein 1
GGA1	Q9UJY5	ADP-ribosylation factor-binding protein GGA1
MCM7	P33993	DNA replication licensing factor MCM7
RPS16	P62249	40S ribosomal protein S16
LATS1	O95835	Serine/threonine-protein kinase LATS1
CCND1	P24385	G1/S-specific cyclin-D1
SKIL	P12757	Ski-like protein
SALL2	Q9Y467	Sal-like protein 2
FTH1	P02794	Ferritin heavy chain
IFIH1	Q9BYX4	Interferon-induced helicase C domain-containing protein 1
MST1R	Q04912	Macrophage-stimulating protein receptor
HSPA5	P11021	78 kDa glucose-regulated protein
DRG1	Q9Y295	Developmentally-regulated GTP-binding protein 1
UBE3C	Q15386	Ubiquitin-protein ligase E3C
USP1	O94782	Ubiquitin carboxyl-terminal hydrolase 1
HERC3	Q15034	Probable E3 ubiquitin-protein ligase HERC3
PSMD6	Q15008	26S proteasome non-ATPase regulatory subunit 6
EPOR	P19235	Erythropoietin receptor
ESR1	P03372	Estrogen receptor
AIMP2	Q13155	Aminoacyl tRNA synthase complex-interacting multifunctional protein 2
CDC25C	P30307	M-phase inducer phosphatase 3

HGS	O14964	Hepatocyte growth factor-regulated tyrosine kinase substrate
USP21	Q9UK80	Ubiquitin carboxyl-terminal hydrolase 21
TP63	Q9H3D4	Tumor protein 63
COMMD1	Q8N668	COMM domain-containing protein 1
SMAD1	Q15797	Mothers against decapentaplegic homolog 1
ADRB2	P07550	Beta-2 adrenergic receptor
ARRB2	P32121	Beta-arrestin-2
TBK1	Q9UHD2	Serine/threonine-protein kinase TBK1
RIPK2	O43353	Receptor-interacting serine/threonine-protein kinase 2
PSMA4	P25789	Proteasome subunit alpha type-4
NEDD8	Q15843	NEDD8
NFKBIA	P25963	NF-kappa-B inhibitor alpha
FANCD2	Q9BXW9	Fanconi anemia group D2 protein
PML	P29590	Protein PML
CKB	P12277	Creatine kinase B-type
WWP1	Q9H0M0	NEDD4-like E3 ubiquitin-protein ligase WWP1
CDT1	Q9H211	DNA replication factor Cdt1
KRT8	P05787	Keratin, type II cytoskeletal 8
PTK2	Q05397	Focal adhesion kinase 1
HSPA4	P34932	Heat shock 70 kDa protein 4
APP	P05067	Amyloid beta A4 protein
TRIM25	Q14258	E3 ubiquitin/ISG15 ligase TRIM25
RPS27A	P62979	Ubiquitin-40S ribosomal protein S27a
TRAF3	A6NHG8	TNF receptor-associated factor 3
AXIN1	O15169	Axin-1
DUSP1	P28562	Dual specificity protein phosphatase 1
RBL2	Q08999	Retinoblastoma-like protein 2
POLR2A	P24928	DNA-directed RNA polymerase II subunit RPB1
CDK5RAP3	Q96JB5	CDK5 regulatory subunit-associated protein 3
USP7	Q93009	Ubiquitin carboxyl-terminal hydrolase 7

UCHL1	P09936	Ubiquitin carboxyl-terminal hydrolase isozyme L1
AKT1	P31749	RAC-alpha serine/threonine-protein kinase
HSPD1	P10809	60 kDa heat shock protein, mitochondrial
CD4	P01730	T-cell surface glycoprotein CD4
RPS7	P62081	40S ribosomal protein S7
ERBB2	P04626	Receptor tyrosine-protein kinase erbB-2
YWHAE	P62258	14-3-3 protein epsilon
PIK3R1	P27986	Phosphatidylinositol 3-kinase regulatory subunit alpha
NEUROD1	Q13562	Neurogenic differentiation factor 1
SNCAIP	Q9Y6H5	Synphilin-1
CYLD	Q9NQC7	Ubiquitin carboxyl-terminal hydrolase CYLD
COPE	O14579	Coatomer subunit epsilon
FASN	P49327	Fatty acid synthase
UNG	P13051	Uracil-DNA glycosylase
TRAF2	Q12933	TNF receptor-associated factor 2
LAPTM5	Q13571	Lysosomal-associated transmembrane protein 5
UBE2S	Q16763	Ubiquitin-conjugating enzyme E2 S
PSMC1	P62191	26S protease regulatory subunit 4
UBE2G2	P60604	Ubiquitin-conjugating enzyme E2 G2
NDFIP2	Q9NV92	NEDD4 family-interacting protein 2
HNRNPA1	P09651	Heterogeneous nuclear ribonucleoprotein A1
DDB1	Q16531	DNA damage-binding protein 1
SOD2	A0A0C4DFU1	Superoxide dismutase
USP15	Q9Y4E8	Ubiquitin carboxyl-terminal hydrolase 15
MSH6	P52701	DNA mismatch repair protein Msh6
SCNN1G	P51170	Amiloride-sensitive sodium channel subunit gamma
RARRES3	Q9UL19	Retinoic acid receptor responder protein 3
IQGAP1	P46940	Ras GTPase-activating-like protein IQGAP1
CCT8	P50990	T-complex protein 1 subunit theta
UBXN1	Q04323	UBX domain-containing protein 1

TSG101	Q99816	Tumor susceptibility gene 101 protein
SNRNP200	O75643	U5 small nuclear ribonucleoprotein 200 kDa helicase
HUWE1	Q7Z6Z7	E3 ubiquitin-protein ligase HUWE1
ERBB4	Q15303	Receptor tyrosine-protein kinase erbB-4
CBLB	Q13191	E3 ubiquitin-protein ligase CBL-B
NOTCH1	P46531	Neurogenic locus notch homolog protein 1
HLTF	Q14527	Helicase-like transcription factor
PSMD7	P51665	26S proteasome non-ATPase regulatory subunit 7
STAM2	O75886	Signal transducing adapter molecule 2
PSMC4	P43686	26S protease regulatory subunit 6B
NTRK2	Q16620	BDNF/NT-3 growth factors receptor
RPL6	Q02878	60S ribosomal protein L6
ATF4	P18848	Cyclic AMP-dependent transcription factor ATF-4
IRF8	Q02556	Interferon regulatory factor 8
UBASH3B	Q8TF42	Ubiquitin-associated and SH3 domain-containing protein B
CDKN1B	P46527	Cyclin-dependent kinase inhibitor 1B
NF2	P35240	Merlin
HNRNPD	Q14103	Heterogeneous nuclear ribonucleoprotein D0
PSMD1	Q99460	26S proteasome non-ATPase regulatory subunit 1
PGAM5	Q96HS1	Serine/threonine-protein phosphatase PGAM5, mitochondrial
GGA3	Q9NZ52	ADP-ribosylation factor-binding protein GGA3
HSPA2	P54652	Heat shock-related 70 kDa protein 2
INSR	P06213	Insulin receptor
EEF1A1	P68104	Elongation factor 1-alpha 1
SCNN1A	P37088	Amiloride-sensitive sodium channel subunit alpha
TNIP2	Q8NFZ5	TNFAIP3-interacting protein 2
UBE2L3	P68036	Ubiquitin-conjugating enzyme E2 L3
USP11	P51784	Ubiquitin carboxyl-terminal hydrolase 11
CLTC	Q00610	Clathrin heavy chain 1
UBD	O15205	Ubiquitin D

		UBE2C	O00762	Ubiquitin-conjugating enzyme E2 C
		UBE2D1	P51668	Ubiquitin-conjugating enzyme E2 D1
		UBE2D2	P62837	Ubiquitin-conjugating enzyme E2 D2
		UBE2D3	P61077	Ubiquitin-conjugating enzyme E2 D3
		UBE2T	Q9NPD8	Ubiquitin-conjugating enzyme E2 T
		UBE3A	Q05086	Ubiquitin-protein ligase E3A
		UBQLN1	Q9UMX0	Ubiquilin-1
		UBQLN2	Q9UHD9	Ubiquilin-2
		UBQLN4	Q9NRR5	Ubiquilin-4
		UCHL3	P15374	Ubiquitin carboxyl-terminal hydrolase isozyme L3
		UCHL5	Q9Y5K5	Ubiquitin carboxyl-terminal hydrolase isozyme L5
		UIMC1	Q96RL1	BRCA1-A complex subunit RAP80
		VCP	P55072	Transitional endoplasmic reticulum ATPase
		VPS36	Q86VN1	Vacuolar protein-sorting-associated protein 36
		WWP2	O00308	NEDD4-like E3 ubiquitin-protein ligase WWP2
		XIAP	P98170	E3 ubiquitin-protein ligase XIAP
		XRCC6	P12956	X-ray repair cross-complementing protein 6
		YBX1	P67809	Nuclease-sensitive element-binding protein 1
		ZRANB1	Q9UGI0	Ubiquitin thioesterase ZRANB1
PSEN1	P49768	APH1A	Q96BI3	Gamma-secretase subunit APH-1A
		CTNNB1	P35222	Catenin beta-1
		CDH1	P12830	Cadherin-1
		PSENEN	Q9NZ42	Gamma-secretase subunit PEN-2
		KCNIP3	Q9Y2W7	Calsenilin
		APP	P05067	Amyloid beta A4 protein
		GSK3B	P49841	Glycogen synthase kinase-3 beta
		NCSTN	Q92542	Nicastrin
		CTNND2	Q9UQB3	Catenin delta-2
		UBQLN1	Q9UMX0	Ubiquilin-1
NR5A2	O00482	NR0B2	Q15466	Nuclear receptor subfamily 0 group B member 2

		CTNNB1	P35222	Catenin beta-1
TRRAP	Q9Y4A5	CTNNB1	P35222	Catenin beta-1
		ESR1	P03372	Estrogen receptor
		KAT2A	Q92830	Histone acetyltransferase KAT2A
		KAT2B	Q92831	Histone acetyltransferase KAT2B
		KAT5	Q92993	Histone acetyltransferase KAT5
		TAF10	Q12962	Transcription initiation factor TFIID subunit 10
		MYC	P01106	Myc proto-oncogene protein
		MAX	P61244	Protein max
		ATXN7	O15265	Ataxin-7
AXIN1	O15169	LRP6	O75581	Low-density lipoprotein receptor-related protein 6
		APC	P25054	Adenomatous polyposis coli protein
		CSNK1A1	P48729	Casein kinase I isoform alpha
		AXIN2	Q9Y2T1	Axin-2
		CSNK1E	P49674	Casein kinase I isoform epsilon
		CTNNB1	P35222	Catenin beta-1
		DVL1	O14640	Segment polarity protein dishevelled homolog DVL-1
		DVL3	Q92997	Segment polarity protein dishevelled homolog DVL-3
		GSK3B	P49841	Glycogen synthase kinase-3 beta
		MAP3K1	Q13233	Mitogen-activated protein kinase kinase kinase 1
		PPP2CA	P67775	Serine/threonine-protein phosphatase 2A catalytic subunit alpha isoform
		UBC	P0CG48	Polyubiquitin-C
HDAC1	Q13547	SUV39H1	O43463	Histone-lysine N-methyltransferase SUV39H1
		SIN3B	O75182	Paired amphipathic helix protein Sin3b
		RELA	Q04206	Transcription factor p65
		RBBP7	Q16576	Histone-binding protein RBBP7
		MYC	P01106	Myc proto-oncogene protein
		MTA3	Q9BTC8	Metastasis-associated protein MTA3
		MECP2	P51608	Methyl-CpG-binding protein 2
		MDM2	J3KN53	E3 ubiquitin-protein ligase Mdm2

ING1	A0A0C4DFW2	Inhibitor of growth protein
HDAC9	Q9UKV0	Histone deacetylase 9
RBL2	Q08999	Retinoblastoma-like protein 2
ZBTB16	Q05516	Zinc finger and BTB domain-containing protein 16
RUNX1T1	Q06455	Protein CBFA2T1
SPI1	P17947	Transcription factor PU.1
SMAD3	P84022	Mothers against decapentaplegic homolog 3
TGIF1	Q15583	Homeobox protein TGIF1
SAP30	O75446	Histone deacetylase complex subunit SAP30
E2F1	Q01094	Transcription factor E2F1
CHD4	Q14839	Chromodomain-helicase-DNA-binding protein 4
MYOD1	P15172	Myoblast determination protein 1
KDM1A	O60341	Lysine-specific histone demethylase 1A
TAL1	P17542	T-cell acute lymphocytic leukemia protein 1
SNAI1	O95863	Zinc finger protein SNAI1
MBD2	Q9UBB5	Methyl-CpG-binding domain protein 2
SIN3A	Q96ST3	Paired amphipathic helix protein Sin3a
NCOR2	Q9Y618	Nuclear receptor corepressor 2
STAT3	P40763	Signal transducer and activator of transcription 3
RUNX1	Q01196	Runt-related transcription factor 1
PHB	P35232	Prohibitin
RB1	P06400	Retinoblastoma-associated protein
NONO	Q15233	Non-POU domain-containing octamer-binding protein
RCOR1	J3KN32	REST corepressor 1
EED	O75530	Polycomb protein EED
SP1	P08047	Transcription factor Sp1
DNMT1	P26358	DNA (cytosine-5)-methyltransferase 1
RUVBL2	X6R2L4	RuvB-like 2
MIER1	Q8N108	Mesoderm induction early response protein 1
UBC	P0CG48	Polyubiquitin-C

WHSC1	O96028	Histone-lysine N-methyltransferase NSD2
RUNX2	Q13950	Runt-related transcription factor 2
DDX5	P17844	Probable ATP-dependent RNA helicase DDX5
PML	P29590	Protein PML
CSNK2A1	P68400	Casein kinase II subunit alpha
DNMT3B	Q9UBC3	DNA (cytosine-5)-methyltransferase 3B
SMAD2	Q15796	Mothers against decapentaplegic homolog 2
NR0B2	Q15466	Nuclear receptor subfamily 0 group B member 2
GATAD2B	Q8WXI9	Transcriptional repressor p66-beta
RBBP4	Q09028	Histone-binding protein RBBP4
NFKB1	P19838	Nuclear factor NF-kappa-B p105 subunit
HIF1A	Q16665	Hypoxia-inducible factor 1-alpha
KDM5B	Q9UGL1	Lysine-specific demethylase 5B
SFPQ	P23246	Splicing factor, proline- and glutamine-rich
CTBP1	Q13363	C-terminal-binding protein 1
MTA2	O94776	Metastasis-associated protein MTA2
CTNNB1	P35222	Catenin beta-1
SATB1	Q01826	DNA-binding protein SATB1
MBD3	O95983	Methyl-CpG-binding domain protein 3
EZH2	Q15910	Histone-lysine N-methyltransferase EZH2
SMARCA4	P51532	Transcription activator BRG1
KAT5	Q92993	Histone acetyltransferase KAT5
YY1	P25490	Transcriptional repressor protein YY1
MTA1	Q13330	Metastasis-associated protein MTA1
TP53	P04637	Cellular tumor antigen p53
SP3	Q02447	Transcription factor Sp3
EP300	Q09472	Histone acetyltransferase p300
ESR1	P03372	Estrogen receptor
PPP1CC	P36873	Serine/threonine-protein phosphatase PP1-gamma catalytic subunit
SAP130	Q9H0E3	Histone deacetylase complex subunit SAP130

		ING2	Q9H160	Inhibitor of growth protein 2
		BCL6	P41182	B-cell lymphoma 6 protein
		CHD3	Q12873	Chromodomain-helicase-DNA-binding protein 3
		BRMS1	G5E9I4	Breast cancer metastasis suppressor 1, isoform CRA_c
		AR	A0A0B4J1T2	Androgen receptor
GSK3B	P49841	CTNNB1	P35222	Catenin beta-1
		FRAT1	Q92837	Proto-oncogene FRAT1
		AXIN2	Q9Y2T1	Axin-2
		AKT1	P31749	RAC-alpha serine/threonine-protein kinase
		NIN	Q8N4C6	Ninein
		SNAI1	O95863	Zinc finger protein SNAI1
		AXIN1	O15169	Axin-1
		SGK3	Q96BR1	Serine/threonine-protein kinase Sgk3
		TP53	P04637	Cellular tumor antigen p53
		MAPT	P10636	Microtubule-associated protein tau
		MYC	P01106	Myc proto-oncogene protein
		PSEN1	P49768	Presenilin-1
TCF4	P15884	ID2	Q02363	DNA-binding protein inhibitor ID-2
		TAL1	P17542	T-cell acute lymphocytic leukemia protein 1
		ID3	Q02535	DNA-binding protein inhibitor ID-3
		CTNNB1	P35222	Catenin beta-1
		ID1	P41134	DNA-binding protein inhibitor ID-1
CUL1	Q13616	BTRC	Q9Y297	F-box/WD repeat-containing protein 1A
		COPS5	Q92905	COP9 signalosome complex subunit 5
		CTNNB1	P35222	Catenin beta-1
		FBXO7	Q9Y3I1	F-box only protein 7
		CAND1	Q86VP6	Cullin-associated NEDD8-dissociated protein 1
		NEDD8	Q15843	NEDD8
		CHEK1	J3KN87	Serine/threonine-protein kinase Chk1
		COPS6	Q7L5N1	COP9 signalosome complex subunit 6

		DCUN1D1	Q96GG9	DCN1-like protein 1
		FBXW7	Q969H0	F-box/WD repeat-containing protein 7
		FBXW11	Q9UKB1	F-box/WD repeat-containing protein 11
		UBE2M	P61081	NEDD8-conjugating enzyme Ubc12
		CDC34	P49427	Ubiquitin-conjugating enzyme E2 R1
		FBXW8	Q8N3Y1	F-box/WD repeat-containing protein 8
		FBXO4	Q9UKT5	F-box only protein 4
		RBX1	P62877	E3 ubiquitin-protein ligase RBX1
		SKP1	P63208	S-phase kinase-associated protein 1
		SKP2	Q13309	S-phase kinase-associated protein 2
		RNF7	Q9UBF6	RING-box protein 2
		PSMA2	P25787	Proteasome subunit alpha type-2
		NFKBIA	P25963	NF-kappa-B inhibitor alpha
		COPS2	P61201	COP9 signalosome complex subunit 2
		FBXO6	Q9NRD1	F-box only protein 6
		FBXW2	Q9UKT8	F-box/WD repeat-containing protein 2
		GLMN	Q92990	Glomulin
		GPS1	Q13098	COP9 signalosome complex subunit 1
		UBC	P0CG48	Polyubiquitin-C
RUVBL1	Q9Y265	CTNNB1	P35222	Catenin beta-1
		TTI1	O43156	TELO2-interacting protein 1 homolog
		C2orf44	Q9H6R7	WD repeat and coiled-coil-containing protein C2orf44
		RUVBL2	X6R2L4	RuvB-like 2
		DMAP1	Q9NPF5	DNA methyltransferase 1-associated protein 1
		PIH1D1	Q9NWS0	PIH1 domain-containing protein 1
		PRKDC	P78527	DNA-dependent protein kinase catalytic subunit
		INO80E	J3KNE2	INO80 complex subunit E
		MYC	P01106	Myc proto-oncogene protein
		KAT5	Q92993	Histone acetyltransferase KAT5
		ACTL6A	O96019	Actin-like protein 6A

CTNNA1	P35221	CDH5	P33151	Cadherin-5
		VCL	P18206	Vinculin
		APC	P25054	Adenomatous polyposis coli protein
		CDH3	P22223	Cadherin-3
		CDH1	P12830	Cadherin-1
		CTNNB1	P35222	Catenin beta-1
		JUP	P14923	Junction plakoglobin
		PARD3	Q8TEW0	Partitioning defective 3 homolog
CHD8	Q9HCK8	WDR5	P61964	WD repeat-containing protein 5
		CTNNB1	P35222	Catenin beta-1
PTPRF	P10586	CTNNB1	P35222	Catenin beta-1
		PPFIA1	Q13136	Liprin-alpha-1
PARD3	Q8TEW0	PARD6B	Q9BYG5	Partitioning defective 6 homolog beta
		CDH5	P33151	Cadherin-5
		CTNNB1	P35222	Catenin beta-1
		CTNNA1	P35221	Catenin alpha-1
		JAM3	Q9BX67	Junctional adhesion molecule C
		PARD6A	Q9NPB6	Partitioning defective 6 homolog alpha
		JAM2	P57087	Junctional adhesion molecule B
AXIN2	Q9Y2T1	GSK3B	P49841	Glycogen synthase kinase-3 beta
		CTNNB1	P35222	Catenin beta-1
		AXIN1	O15169	Axin-1
EGFR	P00533	STAT3	P40763	Signal transducer and activator of transcription 3
		RASA1	P20936	Ras GTPase-activating protein 1
		PLCG1	P19174	1-phosphatidylinositol 4,5-bisphosphate phosphodiesterase gamma-1
		PIK3R1	P27986	Phosphatidylinositol 3-kinase regulatory subunit alpha
		NCK2	O43639	Cytoplasmic protein NCK2
		LRIG1	Q96JA1	Leucine-rich repeats and immunoglobulin-like domains protein 1
		GAB1	Q13480	GRB2-associated-binding protein 1
		ESR1	P03372	Estrogen receptor

ERBB3	P21860	Receptor tyrosine-protein kinase erbB-3
ERBB2	P04626	Receptor tyrosine-protein kinase erbB-2
SH3KBP1	Q96B97	SH3 domain-containing kinase-binding protein 1
CTNND1	O60716	Catenin delta-1
YWHAZ	P63104	14-3-3 protein zeta/delta
YWHAZ	P63104	14-3-3 protein zeta/delta
GRB2	P62993	Growth factor receptor-bound protein 2
SOS1	Q07889	Son of sevenless homolog 1
AR	A0A0B4J1T2	Androgen receptor
UBC	P0CG48	Polyubiquitin-C
PTPN11	Q06124	Tyrosine-protein phosphatase non-receptor type 11
TGFA	P01135	Protransforming growth factor alpha
CTNNB1	P35222	Catenin beta-1
PTPRJ	Q12913	Receptor-type tyrosine-protein phosphatase eta
SHC1	P29353	SHC-transforming protein 1
STAT1	P42224	Signal transducer and activator of transcription 1-alpha/beta
PTPN1	P18031	Tyrosine-protein phosphatase non-receptor type 1
TNK2	Q07912	Activated CDC42 kinase 1
VAV2	P52735	Guanine nucleotide exchange factor VAV2
HGS	O14964	Hepatocyte growth factor-regulated tyrosine kinase substrate
EPS15	P42566	Epidermal growth factor receptor substrate 15
MUC1	P15941	Mucin-1
CALM1	P62158	Calmodulin
SRC	P12931	Proto-oncogene tyrosine-protein kinase Src
NCK1	P16333	Cytoplasmic protein NCK1
EPN1	Q9Y6I3	Epsin-1
JAK2	O60674	Tyrosine-protein kinase JAK2
IGHG1	A0A0A0MS08	Ig gamma-1 chain C region (Fragment)
HSP90AA1	P07900	Heat shock protein HSP 90-alpha
CRK	P46108	Adapter molecule crk

		PDCD6IP	Q8WUM4	Programmed cell death 6-interacting protein
		ERRFI1	Q9UJM3	ERBB receptor feedback inhibitor 1
		CAV1	Q03135	Caveolin-1
		EGF	P01133	Pro-epidermal growth factor
		CBLB	Q13191	E3 ubiquitin-protein ligase CBL-B
		CBL	P22681	E3 ubiquitin-protein ligase CBL
MEN1	O00255	ASH2L	Q9UBL3	Set1/Ash2 histone methyltransferase complex subunit ASH2
		CTNNB1	P35222	Catenin beta-1
		RBBP5	Q15291	Retinoblastoma-binding protein 5
		JUND	P17535	Transcription factor jun-D
TCF7L2	Q9NQB0	CTNNB1	P35222	Catenin beta-1
		EP300	Q09472	Histone acetyltransferase p300
		BCL9	O00512	B-cell CLL/lymphoma 9 protein
FBXW11	Q9UKB1	TUBGCP3	Q96CW5	Gamma-tubulin complex component 3
		SKP1	P63208	S-phase kinase-associated protein 1
		NFKBIA	P25963	NF-kappa-B inhibitor alpha
		HIVEP2	P31629	Transcription factor HIVEP2
		USP47	Q96K76	Ubiquitin carboxyl-terminal hydrolase 47
		CLPX	O76031	ATP-dependent Clp protease ATP-binding subunit clpX-like, mitochondrial
		HIVEP1	P15822	Zinc finger protein 40
		MED20	Q9H944	Mediator of RNA polymerase II transcription subunit 20
		NUDC	Q9Y266	Nuclear migration protein nudC
		IFNAR1	P17181	Interferon alpha/beta receptor 1
		CTNNB1	P35222	Catenin beta-1
		NRD1	B1AKJ5	Nardilysin
		CUL1	Q13616	Cullin-1
		CDC25A	P30304	M-phase inducer phosphatase 1
		BTRC	Q9Y297	F-box/WD repeat-containing protein 1A
		OGT	O15294	UDP-N-acetylglucosamine--peptide N-acetylglucosaminyltransferase 110 kDa subunit

		PDCD4	Q53EL6	Programmed cell death protein 4
		CDC25B	P30305	M-phase inducer phosphatase 2
CTNNBIP1	Q9NSA3	CTNNB1	P35222	Catenin beta-1
CDH5	P33151	KDR	P35968	Vascular endothelial growth factor receptor 2
		F2RL2	O00254	Proteinase-activated receptor 3
		JUP	P14923	Junction plakoglobin
		PARD6A	Q9NPB6	Partitioning defective 6 homolog alpha
		PARD6G	Q9BYG4	Partitioning defective 6 homolog gamma
		CTNNA1	P35221	Catenin alpha-1
		CTNNB1	P35222	Catenin beta-1
		PARD3	Q8TEW0	Partitioning defective 3 homolog
APC	P25054	CTNNA1	P35221	Catenin alpha-1
		AXIN1	O15169	Axin-1
		BTRC	Q9Y297	F-box/WD repeat-containing protein 1A
		JUP	P14923	Junction plakoglobin
		UBC	P0CG48	Polyubiquitin-C
		CTNNB1	P35222	Catenin beta-1
		ARHGEF4	Q9NR80	Rho guanine nucleotide exchange factor 4
		MAPRE1	Q15691	Microtubule-associated protein RP/EB family member 1
SKP1	P63208	FBXO11	A0A0A0MR53	F-box only protein 11
		CKS1B	P61024	Cyclin-dependent kinases regulatory subunit 1
		FBXO2	Q9UK22	F-box only protein 2
		BTRC	Q9Y297	F-box/WD repeat-containing protein 1A
		ZC3HC1	Q86WB0	Nuclear-interacting partner of ALK
		FBXW11	Q9UKB1	F-box/WD repeat-containing protein 11
		FBXL2	Q9UKC9	F-box/LRR-repeat protein 2
		FBXO44	Q9H4M3	F-box only protein 44
		FBXO4	Q9UKT5	F-box only protein 4
		SKP2	Q13309	S-phase kinase-associated protein 2
		FBXW8	Q8N3Y1	F-box/WD repeat-containing protein 8

		RBX1	P62877	E3 ubiquitin-protein ligase RBX1
		NFKBIA	P25963	NF-kappa-B inhibitor alpha
		FBXO7	Q9Y3I1	F-box only protein 7
		CTNNB1	P35222	Catenin beta-1
		MYC	P01106	Myc proto-oncogene protein
		CUL1	Q13616	Cullin-1
		FBXW2	Q9UKT8	F-box/WD repeat-containing protein 2
		FBXL5	Q9UKA1	F-box/LRR-repeat protein 5
		FBXO6	Q9NRD1	F-box only protein 6
		FBXW7	Q969H0	F-box/WD repeat-containing protein 7
		FBXW5	Q969U6	F-box/WD repeat-containing protein 5
CREBBP	Q92793	TP53	P04637	Cellular tumor antigen p53
		STAT6	P42226	Signal transducer and activator of transcription 6
		STAT3	P40763	Signal transducer and activator of transcription 3
		STAT1	P42224	Signal transducer and activator of transcription 1-alpha/beta
		SREBF1	P36956	Sterol regulatory element-binding protein 1
		SMARCA4	P51532	Transcription activator BRG1
		SMAD3	P84022	Mothers against decapentaplegic homolog 3
		SMAD1	Q15797	Mothers against decapentaplegic homolog 1
		RELA	Q04206	Transcription factor p65
		PML	P29590	Protein PML
		NFE2L2	Q16236	Nuclear factor erythroid 2-related factor 2
		NCOA6	Q14686	Nuclear receptor coactivator 6
		NCOA3	Q9Y6Q9	Nuclear receptor coactivator 3
		NCOA2	Q15596	Nuclear receptor coactivator 2
		NCOA1	Q15788	Nuclear receptor coactivator 1
		MYB	P10242	Transcriptional activator Myb
		MDM2	J3KN53	E3 ubiquitin-protein ligase Mdm2
		KLF4	O43474	Krueppel-like factor 4

		KHDRBS1	Q07666	KH domain-containing, RNA-binding, signal transduction-associated protein 1
		KAT2B	Q92831	Histone acetyltransferase KAT2B
		JUN	P05412	Transcription factor AP-1
		IRF3	Q14653	Interferon regulatory factor 3
		HTT	P42858	Huntingtin
		HNF4A	P41235	Hepatocyte nuclear factor 4-alpha
		HMGA1	P17096	High mobility group protein HMG-I/HMG-Y
		HIF1A	Q16665	Hypoxia-inducible factor 1-alpha
		GTF2B	Q00403	Transcription initiation factor IIB
		FOXO4	P98177	Forkhead box protein O4
		FOXO1	Q12778	Forkhead box protein O1
		ETS2	P15036	Protein C-ets-2
		ETS1	P14921	Protein C-ets-1
		EP300	Q09472	Histone acetyltransferase p300
		CTNNB1	P35222	Catenin beta-1
		VDR	P11473	Vitamin D3 receptor
		MYOD1	P15172	Myoblast determination protein 1
		MYBL2	P10244	Myb-related protein B
		SMAD2	Q15796	Mothers against decapentaplegic homolog 2
		NR3C1	P04150	Glucocorticoid receptor
		ESR1	P03372	Estrogen receptor
		CREB1	P16220	Cyclic AMP-responsive element-binding protein 1
		CARM1	Q86X55	Histone-arginine methyltransferase CARM1
		BRCA1	P38398	Breast cancer type 1 susceptibility protein
		ATF2	P15336	Cyclic AMP-dependent transcription factor ATF-2
		AR	A0A0B4J1T2	Androgen receptor
CDH1	P12830	SKP2	Q13309	S-phase kinase-associated protein 2
		PSEN1	P49768	Presenilin-1
		CTNND1	O60716	Catenin delta-1

		JUP	P14923	Junction plakoglobin
		SRC	P12931	Proto-oncogene tyrosine-protein kinase Src
		UBC	P0CG48	Polyubiquitin-C
		CBLL1	Q75N03	E3 ubiquitin-protein ligase Hakai
		CTNNA1	P35221	Catenin alpha-1
		KLRG1	Q96E93	Killer cell lectin-like receptor subfamily G member 1
		CTNNB1	P35222	Catenin beta-1
RUVBL2	X6R2L4	CTNNB1	P35222	Catenin beta-1
		KAT5	Q92993	Histone acetyltransferase KAT5
		INO80E	J3KNE2	INO80 complex subunit E
		RUVBL1	Q9Y265	RuvB-like 1
		HDAC1	Q13547	Histone deacetylase 1
		YY1	P25490	Transcriptional repressor protein YY1
		ACTL6A	O96019	Actin-like protein 6A
		DPCD	Q9BVM2	Protein DPCD
		C2orf44	Q9H6R7	WD repeat and coiled-coil-containing protein C2orf44
		TTI1	O43156	TELO2-interacting protein 1 homolog
		MYC	P01106	Myc proto-oncogene protein
		DMAP1	Q9NPF5	DNA methyltransferase 1-associated protein 1
APC2	O95996	CTNNB1	P35222	Catenin beta-1
ERBB2	P04626	CTNNB1	P35222	Catenin beta-1
		HSP90AA1	P07900	Heat shock protein HSP 90-alpha
		GRB7	Q14451	Growth factor receptor-bound protein 7
		ERBB4	Q15303	Receptor tyrosine-protein kinase erbB-4
		SHC1	P29353	SHC-transforming protein 1
		PIK3R1	P27986	Phosphatidylinositol 3-kinase regulatory subunit alpha
		GRB2	P62993	Growth factor receptor-bound protein 2
		EGFR	P00533	Epidermal growth factor receptor
		ERBB2IP	Q96RT1	Protein LAP2
		IGHG1	A0A0A0MS08	Ig gamma-1 chain C region (Fragment)

		UBC	P0CG48	Polyubiquitin-C
		SRC	P12931	Proto-oncogene tyrosine-protein kinase Src
		HSPA4	P34932	Heat shock 70 kDa protein 4
		ERBB3	P21860	Receptor tyrosine-protein kinase erbB-3
		PLCG1	P19174	1-phosphatidylinositol 4,5-bisphosphate phosphodiesterase gamma-1
		STAT3	P40763	Signal transducer and activator of transcription 3
		STUB1	Q9UNE7	E3 ubiquitin-protein ligase CHIP
LEF1	Q9UJU2	CTNNB1	P35222	Catenin beta-1
BCL9	O00512	PYGO1	Q9Y3Y4	Pygopus homolog 1
		CTNNB1	P35222	Catenin beta-1
		TCF7L2	Q9NQB0	Transcription factor 7-like 2
		PYGO2	Q9BRQ0	Pygopus homolog 2
CDH3	P22223	CTNNB1	P35222	Catenin beta-1
		CTNNA1	P35221	Catenin alpha-1
TCF7	P36402	CTNNB1	P35222	Catenin beta-1
CDH2	P19022	CTNND1	O60716	Catenin delta-1
		JUP	P14923	Junction plakoglobin
		CTNNB1	P35222	Catenin beta-1
PITX2	Q99697	CTNNB1	P35222	Catenin beta-1
RBBP5	Q15291	SETD1A	O15047	Histone-lysine N-methyltransferase SETD1A
		MEN1	O00255	Menin
		WDR5	P61964	WD repeat-containing protein 5
		NCOA6	Q14686	Nuclear receptor coactivator 6
		CXXC1	Q9P0U4	CXXC-type zinc finger protein 1
		CTNNB1	P35222	Catenin beta-1
		KDM6A	O15550	Lysine-specific demethylase 6A
		PAXIP1	Q6ZW49	PAX-interacting protein 1
		ASH2L	Q9UBL3	Set1/Ash2 histone methyltransferase complex subunit ASH2
